# Inoculation of mother’s own milk could personalize pasteurized donor human milk used for feeding preterm infants

**DOI:** 10.1186/s12967-021-03096-7

**Published:** 2021-10-09

**Authors:** D. Mallardi, C. Tabasso, P. Piemontese, S. Morandi, T. Silvetti, F. Biscarini, P. Cremonesi, B. Castiglioni, V. Pica, M. Stuknyte, I. De Noni, O. Amato, N. Liotto, F. Mosca, P. Roggero

**Affiliations:** 1 Neonatal Intensive Care Unit, Fondazione IRCCS Ca’ Granda Ospedale Maggiore Policlinico, Via della Commenda 12, 20122 Milan, Italy; 2 Department of Clinical Sciences and Community Health, University of Milan, Milan, Italy; 3 Institute of Sciences of Food Production (ISPA), National Research Council (CNR), Via Celoria 2, 20133 Milan, Italy; 4 Institute of Agricultural Biology and Biotechnology (IBBA), National Research Council (CNR), U.O.S. Di Lodi, Via Einstein, 26900 Lodi, Italy; 5 Department of Food, Environmental and Nutritional Sciences, University of Milan, Via Celoria 2, 20133 Milan, Italy

**Keywords:** Donor human milk, Mother’s own milk, Preterm infants, Bacterial growth, Human milk microbiome, Peptidomic profile

## Abstract

**Background:**

Human milk is a vehicle for bioactive compounds and beneficial bacteria which promote the establishment of a healthy gut microbiome of newborns, especially of preterm infants. Pasteurized donor human milk (PDHM) is the second-best option when preterm mother’s own milk is unavailable. Since pasteurization affect the microbiological quality of donor milk, PDHM was inoculated with different preterm milk samples and then incubated, in order to evaluate the effect in terms of bacterial growth, human milk microbiome and proteolytic phenomena.

**Methods:**

In an in-vitro study PDHM was inoculated at 10% v/v using ten preterm milk samples. Microbiological, metataxonomic and peptidomic analyses, on preterm milk samples at the baseline (T0), on PDHM and on inoculated milk (IM) samples at T0, after 2 h (T1) and 4 h (T2) of incubation at 37 °C, were conducted.

**Results:**

IM samples at T2 showed a Total Bacterial Count not significantly different (p > 0.01) compared to preterm milk samples. At T2 lactic acid bacteria level was restored in all IM. After inoculation, metataxonomic analysis in IM samples showed that Proteobacteria remained the predominant phylum while Firmicutes moved from 3% at T1 to 9.4% at T2. Peptidomic profile of IM resembled that of PDHM, incubated for the same time, in terms of number and type of peptides.

**Conclusion:**

The study demonstrated that inoculation of PDHM with mother’s own milk could restore bacterial growth and personalize human milk microbiome in PDHM. This effect could be beneficial because of the presence of maternal probiotic bacteria which make PDHM more similar to mother’s own milk.

**Supplementary Information:**

The online version contains supplementary material available at 10.1186/s12967-021-03096-7.

## Background

Human milk (HM) represents the optimal feeding for infants, not only for its nutritional quality, but also for being a rich source of several bioactive compounds and specific peptides, essential for the development of the newborn’s immature immune and digestive systems [1]. Different culture -dependent and -independent methods indicated the presence in HM of a complex community of bacteria which comprises many viable commensals, mutualistic or potentially probiotic bacteria such as Bifidobacteria, Lactic acid bacteria (LAB) and other genera as *Staphylococcus*, *Enterococcus* and *Streptococcus*. These indigenous bacteria, supported by the entero-mammary translocation, characterize the HM microbiome which can be affected by several maternal and environmental factors. In fact, maternal diet and lifestyle, medical history, mode of delivery and milk handling are some of the factors that can contribute to shape HM microbiome composition, leading to an high inter-individual variability of microbial species [2, 3]. HM microbiome plays a beneficial role for the breastfed infants: it promotes the intestinal immune system maturation, digestion and nutrients absorption and inhibition of pathogenic bacteria [4]. The constant intake, during lactation, of beneficial bacteria present in the HM promotes the establishment of an healthy gut microbiome of newborns. The healthy gut microbiota could limit the growth of potential pathogens, by exerting a protective and nutritive role, especially for preterm infants [5, 6].

Mothers who delivered prematurely, often experience significant difficulties in breastfeeding their infants: in fact, many mothers of very preterm infants are able to express only small volumes of their own milk [7]. Given the healthy benefits attributable to human milk, when mother’s own milk is not available or insufficient, donor HM is the second-best option [8].

As recommended by current international guidelines [9, 10], donor HM must be pasteurized, using the Holder method, in order to destroy potential pathogenic microorganisms and to inhibit whatever type of bacterial growth. Although it is important to guarantee microbiological safety, Holder pasteurization leads to a total or partial decrease in the biological quality and effectiveness of pasteurized donor HM (PDHM), inactivating most of the valuable HM microbiota species [11]. Indeed, the probiotic effect of PDHM is not comparable to the probiotic effect of mother’s own milk [12]. Moreover, HM microbial species hydrolyze milk proteins, through to the potential action of bacterial proteases/peptidases. Other proteolytic phenomena can occur upon the action of the endogenous proteases: some of these originates within the mammary gland and acts during the whole lactation period. Therefore, both HM endogenous enzymes and microbiota can release peptides or degrade specific individual milk proteins with several functional properties [13].

Recently, it has been reported that incubating donor HM with preterm mother’s own milk (preterm milk, PM) may be effective in improving the biological quality of PDHM by restoring the original milk microbiome [5]. Thus, in an in-vitro study, PDHM was inoculated with different PM samples and then incubated, in order to evaluate the effect in terms of bacterial growth, HM microbiome and proteolytic phenomena.

## Methods

### Experimental design

**Fig. 1:**
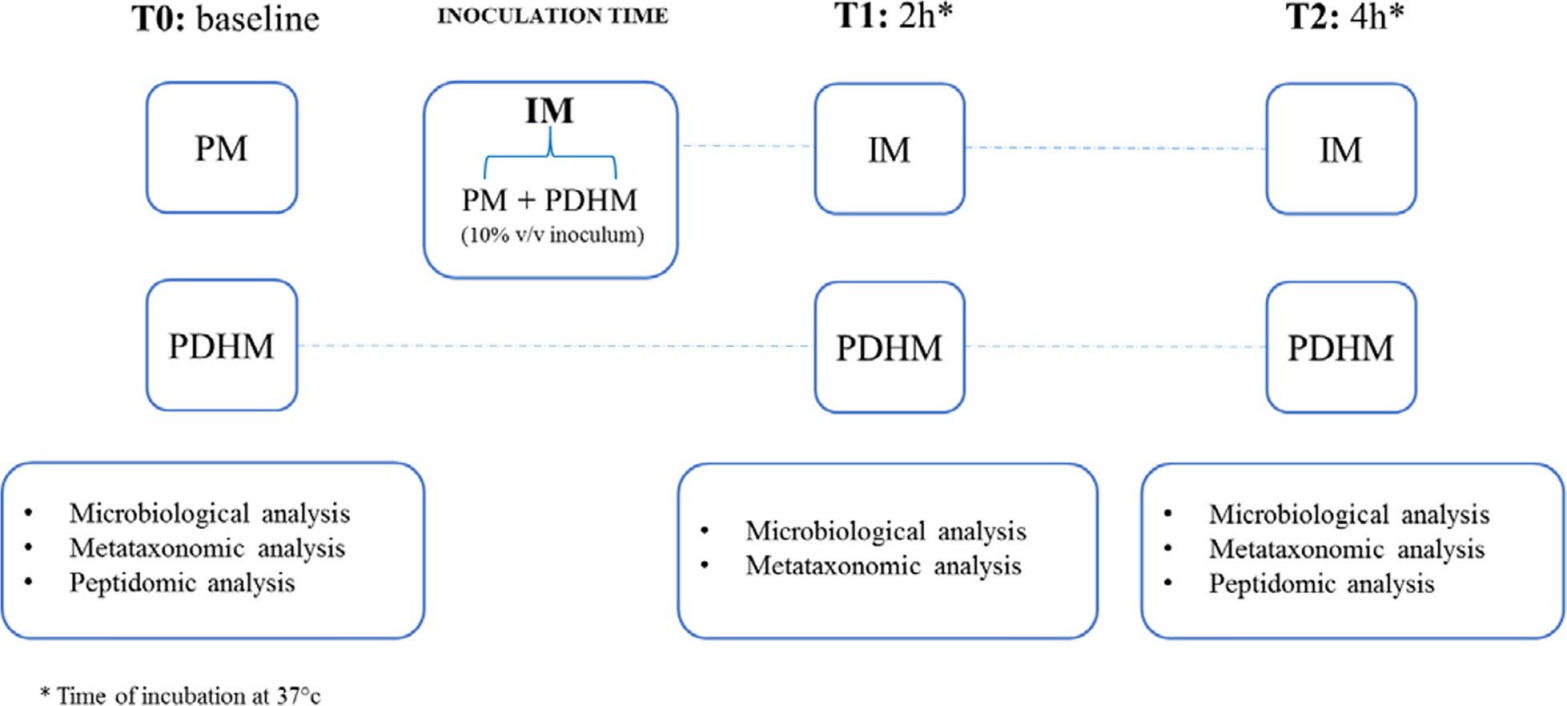
Experimental design of the study. *PM* preterm milk samples, *PDHM* pasteurized donor human milk samples, *IM* inoculated milk samples. T0: baseline (before inoculum); T1: 2 h after inoculation; T2: 4 h after inoculation

Ten samples of inoculated PDHM (hereafter named as IM1 to IM10) were obtained by adding ten different fresh PM samples (hereafter named as PM1 to PM10) at 10% (v/v). Then, the IM samples were incubated at 37 °C for 4 h. The PDHM and IM samples were collected and analyzed at different time points: at the baseline (T0), and 2 h (T1) and 4 h (T2) after inoculation. The adopted experimental design is summarized in Fig. 1. Microbiological and metataxonomic analyses were performed at baseline on PDHM and PM samples and at T1 and T2 on PDHM and IM samples. On the same samples, except for T1, a peptidomic analysis was conducted.

### Subjects

Mothers who delivered prematurely between November 2018 and January 2019 at the Neonatal Intensive Care Unit (NICU) of the Fondazione IRCCS Ca’ Granda Ospedale Maggiore Policlinico (Milan, Italy), were included in the study. Eligible criteria were: delivery before the 37th week of gestational age and absence of maternal antibiotic therapy at the time of the milk collection. Basic maternal and infants’ clinical characteristics were collected using the computed medical records.

Term-delivering mothers, included in this study, donated their milk to the HMB of the Fondazione IRCCS Ca’ Granda Ospedale Maggiore Policlinico, according to HMB Italian guidelines [14]. Written informed consent was obtained for each participating mother. The study was approved by Ethical Committee of the Fondazione IRCCS Ca’ Granda Ospedale Maggiore Policlinico (Approval No. 289_2017).

### Preterm mother’s own milk collection

Prior to milk collection, preterm-delivering mothers, had to perform an accurate hand washing, using an hand sanitizer and a breast washing, using exclusively running water in accordance with the NICU internal procedure. Each sample was collected, using an electric breast pump of the hospital breastfeeding room and a personal breast pump kit, into a sterile bottle. Breast pump kit had to be cleaned and sterilized before every collection.

Between the 30th and the 60th day post-delivery, enrolled preterm-delivering mothers collected a sample of 50 mL of their own fresh milk. All PM samples were collected during the first hours of the morning in the breastfeeding room of the NICU, then they were immediately refrigerated and delivered to the laboratories for analyses. PM3 and PM5 samples were obtained from the same mother in different days.

### Donor HM collection and pasteurization

DHM was collected at home, from the 15th day until 12 months after delivery, by term delivering mothers, belonging to the HMB, who followed the same personal hygiene and material disinfection instruction given to mothers who delivered preterm impatient infants. Each sample was collected using an electric breast pump and a personal breast pump kit, and it was placed into sterile bottle. Breast pump kit had to be cleaned and sterilized before every collection.

Donor HM was stored at donors’ home in a refrigerator for maximum 24 h and thereafter frozen at – 20 °C. During the transport, donor HM was preserved maintaining the cold chain until the arrive to the HMB.

Donor HM samples used to perform inoculation, were collected by donors in different periods of lactation. These samples were thawed, pooled (total volume of 3 L) and then Holder pasteurized at 62.5 °C for 30 min. PDHM was stored at − 80 °C until inoculation with fresh PM samples.

### Inoculation of PDHM with PM

To perform the inoculation, 90 mL of frozen PDHM was gently thawed and inoculated with 10 mL of each fresh PM sample. The low milk volume expressed by preterm-delivering mothers, which necessarily must be used to breastfeed their babies, did not allow us to consider higher inoculation level.

After inoculation, PDHM and IM samples were kept at 37 °C for 4 h (incubation time).

PDHM and PM were analyzed immediately after their arrival in the laboratory. PDHM and IM samples were analyzed after incubation at different time points too, as described in the experimental design (Fig. 1).

### Enumeration of microorganisms

Serial decimal dilutions of the inoculated aliquots in sterile quarter-strength Ringer’s solution (Scharlab, Barcelona, Spain) were prepared, and the following microbiological determinations were carried out. Mesophilic aerobic bacteria were counted on Petrifilm Aerobic Count Plate (3 M, Minneapolis, MN, USA) after incubation at 30 °C for 72 h (ISO 4833–1:2013). *Enterobacteriaceae* were enumerated on Petrifilm *Enterobacteriaceae* Count Plate (3 M) at 37 °C for 24 h (ISO 21528–1:2017). Coliforms and *Escherichia coli* were determined on Petrifilm *E. coli*/Coliform Count Plate (3 M) at 37 °C for 24–48 h. De Man–Rogosa–Sharpe (MRS) agar (Biolife Italiana, Milan, Italy), M17 agar (Biolife Italiana) and Kanamycin Aesculin Azide (KAA) agar (Scharlab) were used for the enumeration of rod-shaped LAB, cocci LAB (lactococci and streptococci) and enterococci, respectively. MRS agar was incubated at 37 °C for 72 h under anaerobic conditions (AnaerocultA, Merck, Darmstad, Germany), while M17 and KAA agar were kept at 37 °C for 48 h. TOS-propionate agar (Sigma-Aldrich, St. Louis, MO, USA) with MUP selective supplement (Sigma-Aldrich) incubated at 37 °C for 72 h under anaerobic conditions (AnaerocultA, Merck) was used to count *Bifidobacterium* spp. [15], whereas P2 agar (peptone, 5 g; beef extract, 3 g; yeast extract, 5 g; sodium lactate, 1 g; agar, 15 g/L) was used for anaerobic enumeration of *Propionibacterium* spp. Cultivating at 30 °C for 7 days [16]. Chloramphenicol Glucose Yeast Extract agar (Sacco Srl, Cadorago, Italy) after incubation at 25 °C for 5 days was used to culture yeasts [17]. *Pseudomonas* agar (Biolife Italiana) with PP *Pseudomonas* supplement (Biolife Italiana) kept at 30 °C for 48 h [18] and *Bacillus cereus* agar base (PEMBA) agar (Biolife Italiana) with *Bacillus cereus* Antimicrobic Supplement (Biolife Italiana) incubated at 30 °C for 24 h [19] were used for the detection of *Pseudomonas* spp. and *Bacillus cereus*, respectively. Baird Parker (BP) agar (Biolife Italiana) with RPF Supplement (Biolife Italiana) was used for coagulase-positive and negative staphylococci counting after incubation at 37 °C for 48 h [20]. At any sampling time, PDHM sample was also analysed for detection of mesophilic aerobic bacteria, *Enterobacteriaceae*, coliforms, *E. coli*, yeasts, *Pseudomonas* spp., coagulase-positive and negative staphylococci and *B. cereus*.

### Search for *Listeria monocytogenes* and *Pseudomonas aeruginosa*

*Listeria monocytogenes* was searched in all IM and PDHM samples by SureFast^®^ *Listeria monocytogenes* PLUS real-time PCR (RT-PCR) assay (R-Biopharm, Darmstadt, Germany) according to the manufacturer’s instructions. The RT-PCR amplification reactions were performed on an Eco Real-Time PCR System (Illumina, San Diego, CA, USA). Detection of *Pseudomonas aeruginosa* was performed in samples where the presence of *Pseudomonas* spp. had been detected by microbiological cultivation method. The adopted protocol was as follows: ten colonies from PP agar plates were randomly picked and sub-cultured overnight in Brain Heart Infusion (BHI) broth (Scharlab) at 30 °C. After growth, the DNA was extracted by the MicroLYSIS kit (Clent Life Science, Stourbridge, UK). The identification of isolates was carried out using *P. aeruginosa* specific primer as previously reported in Cremonesi et al. [21].

### Search for *Staphylococcus aureus* virulence and enterotoxin genes

*Staphylococcus aureus* virulence and enterotoxin genes were explored in samples found to be contaminated with coagulase-positive staphylococci. The DNA was extracted from 1 mL of breast milk as previously described by Cremonesi et al. [22]. The extracted DNA was amplified by a multiplex PCR for the detection of genes encoding for the coagulase (*coa*) and thermonuclease (*nuc*) regions and for the main staphylococcal enterotoxins (*sea*, *sec*, *sed*, *seg*, *seh*, *sei*, *sej* and *sel*) according to Cremonesi et al. [23].

### Metataxonomic analysis

Five mL of milk sample were centrifuged at 500*g* for 10 min at 4 °C; the supernatant was discarded, and the pellet was washed with one mL of saline solution (0.9% NaCl) and centrifuged at 500*g* for 5 min at 4 °C. The supernatant was discarded, and the bacterial DNA was extracted from the samples as described previously [24], by using a method based on the combination of a chaotropic agent, guanidium thiocyanate, with silica particles, to obtain bacterial cell lysis and nuclease inactivation. DNA quality and quantity were assessed using a NanoDrop ND-1000 spectrophotometer (NanoDrop Technologies, Wilmington, DE, USA). The isolated DNA was then stored at − 20 °C until use.

### Metagenomic library preparation and sequencing

Bacterial DNA was amplified using the primers described by Caporaso et al. [25], which targeted the V3-V4 hypervariable regions of the 16S rRNA gene. All PCR amplifications were performed in 25 μL volumes per sample. A total of 12.5 μL of Phusion High-Fidelity Master Mix 2 × (Thermo Fisher Scientific, Waltham, MA, USA) and 0.2 μL of each primer (100 μM) were added to 2 μL of genomic DNA (5 ng/μL). Amplification was performed in an Applied Biosystem 2700 thermal cycler (Thermo Fisher Scientific) using the amplification cycle as follows: samples were denatured at 98 °C for 30 s, followed by 25 cycles with a denaturing step at 98 °C for 30 s, annealing at 56 °C for 1 min and extension at 72 °C for 1 min, and a final extension at 72 °C for 7 min. Amplicons were cleaned with Agencourt AMPure XP kit (Beckman Coulter, Brea, CA, USA) and libraries were prepared following the 16S Metagenomic Sequencing Library Preparation Protocol (Illumina, San Diego, CA, USA). The libraries obtained were quantified by RT-PCR with KAPA Library Quantification Kit (KapaBiosystems, Cape Town, South Africa), pooled in equimolar proportion and sequenced during a single MiSeq (Illumina) run with 2 × 250-base paired-end reads.

### Bioinformatics and statistical analysis

Demultiplexed paired-end reads from 16S rRNA-gene sequencing were first checked for quality using FastQC [26] for an initial assessment. Forward and reverse paired-end reads were joined into single reads using the C +  + program SeqPrep [27]. After joining, reads were filtered for quality based on: (i) maximum three consecutive low-quality base calls (Phred < 19) allowed; (ii) fraction of consecutive high-quality base calls (Phred > 19) in a read over total read length ≥ 0.75; and (iii) no “N”-labeled bases (missing/uncalled) allowed. Reads that did not match all the above criteria were filtered out. All remaining reads were combined in a single FASTA file for the identification and quantification of OTUs (operational taxonomic units). Reads were aligned against the SILVA closed reference sequence collection release 123, with 97% cluster identity [28, 29] applying the CD-HIT clustering algorithm [30]. A pre-defined taxonomy map of reference sequences to taxonomies was then used for taxonomic identification along the main taxa ranks down to the genus level (domain, phylum, class, order, family, genus). By counting the abundance of each OTU, the OTU table was created and then grouped at each phylogenetic level. OTUs with total counts lower than 10 in fewer than two samples were filtered out. All of the above steps, except the FastQC reads quality check, were performed with the QIIME open-source bioinformatics pipeline for microbiome analysis [31].

The milk microbial diversity was assessed within- (alpha diversity) and across- (beta diversity) samples. All indexes (alpha and beta diversity) were estimated from the complete OTU table (at the OTU level), filtered for OTUs with more than 10 total counts distributed in at least two samples. Besides the number of observed OTUs directly counted from the OTU table, within-sample microbial richness and diversity were estimated using the following indexes: Chao1 and ACE (Abundance-based coverage Estimator) for richness, Shannon, Simpson and Fisher’s alpha for diversity [32–37], Simpson E and Pielou’s J (Shannon’s evenness) for evenness [38]. The across- sample milk microbiota diversity was quantified by calculating Bray–Curtis dissimilarities [39]. Prior to the calculation of the Bray–Curtis dissimilarities, OTU counts were normalized for uneven sequencing depth by cumulative sum scaling CSS [40]. Among groups (PDHM, PM, IM) and pairwise Bray–Curtis dissimilarities at different timepoints were evaluated non-parametrically using the permutational analysis of variance approach (999 permutations; [41]). Details on the calculation of the mentioned alpha- and beta-diversity indexes can be found in Biscarini et al. [42].

Descriptive data related to clinical demographic data of preterm/term—delivering mothers and respective newborns, involved in this study, were reported as mean and standard deviation. Bacterial counts were expressed as mean and standard deviation and the significance level was set at 0.01. Metataxonomic data were reported as mean and relative abundance (%) and the significance level was set at 0.05.

### Peptidomic profiling by UPLC/HR-MS/MS

Peptidomic analyses were performed on 10-kDa-ultrafiltered milk samples using an Acquity UPLC module (Waters, Milford, MA, USA) coupled to a Q Exactive hybrid quadrupole-Orbitrap mass spectrometer (Thermo Fisher Scientific, San Jose, CA, USA) and the peptides were identified using the Proteome Discoverer v1.4 software (Thermo Fisher Scientific). Automatic peak detection was performed setting signal-to-noise ratio to four as suggested by Mangé et al. [43]. The sequences of peptides were identified from MS/MS spectra using SequestHT algorithm [44] against a HM protein library constructed based on results from previous studies [43, 45–48]. A non-specific enzyme cleavage pattern was defined, and 12 missed cleavage sites (maximum allowed for the algorithm) were allowed. No static modifications were set. Phosphorylation of serine and threonine, deamidation of asparagine, glutamine and arginine, oxidation of methionine and cyclisation of an N-terminal glutamine to pyro-glutamic acid were selected as dynamic modifications. Mass error tolerance for precursor ions was 5 ppm and for fragment ions was 0.02 Da. A strict false discovery rate of peptide identification was set (FDR = 0.01).

## Results

Nine preterm-delivering mothers were enrolled in the study and ten samples of fresh PM were collected (PM3 and PM5 were collected by the same mother) from November 2018 to January 2019. Clinical demographic data regarding mothers and respective newborns were reported in Table 1.

**Table 1:** Clinical demographic data regarding preterm-delivering mothers and respective newborns

Milk Sample	Gestational age (week)	Birth weight (g)	Maternal age (years)	Parity
PM1	36	2830	30	Primipara
PM2	35	2005	24	Primipara
PM3	31	1150	44	Primipara
PM4	28	810	47	Multipara
PM5	31	1150	44	Primipara
PM6	29	1480	35	Primipara
PM7	34	1660	33	Multipara
PM8	34	1450	33	Primipara
PM9	31	2050	26	Primipara
PM10	32	975	29	Primipara

Four term-delivering mothers donated milk for this study in November 2018. Mean weight and gestational age at birth of their infants was 3160 ± 970.8 and 38.8 ± 1.3 respectively. Main age of donors enrolled was 29.75 ± 2.5 and two of them were primiparae.

### Microbiological features of PDHM, PM and IM samples at different time points of incubation

**Fig. 2:**
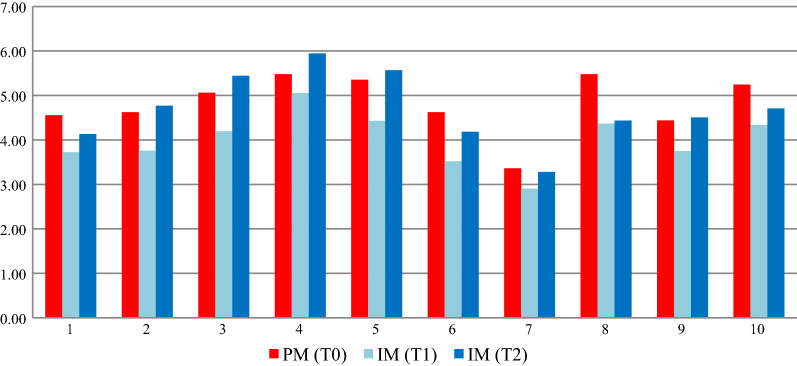
Total bacterial count of PM and IM samples

Total bacterial count (TBC) of PDHM did not change through the incubation with an average load of 2.56 log_10_ CFU/mL, which probably included heat-resistant spore-forming bacteria. Total bacterial count of PM and IM samples is shown in Fig. 2.

A great variability was found among PM samples, which presented a TBC ranging from 3.36 to 5.48 log_10_ CFU/mL. IM samples, although exhibiting different growth evolution, at T2 showed a TBC not significantly different (p > 0.01) from those of PM samples.

The pathogenic bacteria *B. cereus*, *E. coli* and *L. monocytogenes* were not detected. *P. aeruginosa* was revealed only in IM3, IM5 and IM7 samples at T2 (Table 2). In the same samples, *S. aureus* was found at all sampling times (data not shown): specifically at the end of incubation it achieved values comparable to those detected in the corresponding PM samples. Moreover, *S. aureus* strains harboring toxin genes (*sea* and *seh*) were detected in PM3, PM5 and IM3, IM5 samples. Coliforms and *Enterobacteriaceae* in IM3 and IM5 samples were present at similar level (about 2 log_10_ CFU/mL): their count was about 1.5 log higher than in the corresponding PM samples (Table 2).

**Table 2:** Bacterial count of different species of microorganisms in PM and IM samples at T2

Description	Genus/species	Phylum	log_10_ CFU/mL		Samples	
			PM	IM	PM	IM
Pathogens	*B. cereus*	(Fir)	< 1.0	< 1.0	–	–
	*E. coli*	(Pro)	< 1.0	< 1.0	–	–
	*L. monocytogenes*^a^	(Fir)	Absent	Absent	–	–
	*P. aeruginosa*^a^	(Pro)	Absent	Present	–	3, 5, 7
	*S. aureus*	(Fir)	3.8 ± 1.1	4.0 ± 1.3	3, 5, 6, 7	3, 5, 6, 7
	*S. aureus* toxin genes^a^		sea-seh	sea-seh	3, 5	3, 5
Hygiene indicators	Coliforms	(Pro)	2.4 ± 0.8	3.8 ± 0.3	3, 5	3, 5
	*Enterobacteriaceae*	(Pro)	1.9 ± 1.3	4.4 ± 0.9	3, 5	3, 5
	*Pseudomonas* spp.	(Pro)	2.0 ± 1.1	3.4 ± 1.5	3, 5	3, 5, 7
LAB	*Enterococcus* spp.	(Fir)	3.6 ± 1.3	3.8 ± 1.3	4, 10	4, 10
	*Lactobacillus* spp.	(Fir)	3.9 ± 0.8	3.3 ± 1.3	All samples	All samples
	*Streptococcus* spp.	(Fir)	3.3 ± 0.7	3.1 ± 1.8	1, 2, 3, 4, 8, 9, 10	1, 2, 3, 4, 8, 10
Other bacteria	*Bifidobacterium* spp.	(Act)	2.0 ± 0.1	Absent	9	–
	*Propionibacterium* spp.	(Act)	3.2 ± 0.7	2.1 ± 0.6	1, 2, 3, 4, 5, 7	1, 2, 3, 4, 5, 7
Yeasts		(Asc)	2.6 ± 1.1	1.5 ± 0.1	5, 9	5, 9

Data were expressed as mean ± SD

*Fir* firmicutes, *Pro* proteobacteria, *Act* actinobacteria, *Asc* ascomycota

^a^Detected by PCR or RT-PCR

As for LAB, *Lactobacillus* spp. and *Streptococcus* spp. were recovered in many of the PM samples, while enterococci were found in PM4 and PM10. At T2, the corresponding IM samples showed LAB levels not significantly different (p > 0.01). The applied experimental conditions did not promote the growth of Propionibacteria, since their counts in the IM samples were generally 1 log lower than in the PM samples at T2. A similar trend was observed for *Bifidobacterium* and yeasts.

### Metataxonomics analysis of PDHM, PM and IM samples

The microbiota structure of PDHM, PM, and IM samples produced a total of 8,971,219 reads. After quality filtering, 7,934,897 high quality reads were left, with a mean of 152,594 reads per sample (average loss 12.5%). Microbial profiles were evaluated in PDHM, PM and IM samples at different time points. Sequence-based rarefaction curves were obtained from the QIIME pipeline; the sample-based rarefaction curve was produced with ad hoc R functions. The observed number of OTUs detected was plotted as a function of the number of reads (up to 75,000) in each sample and of the number of samples (see Additional file 1: Figure S1). Both curves tend to plateau asymptotically suggesting that sampling and depth of coverage in this analysis were enough to describe the biological diversity of the maternal milk microbiota, although a few samples have been under-sequenced.

**Fig. 3:**
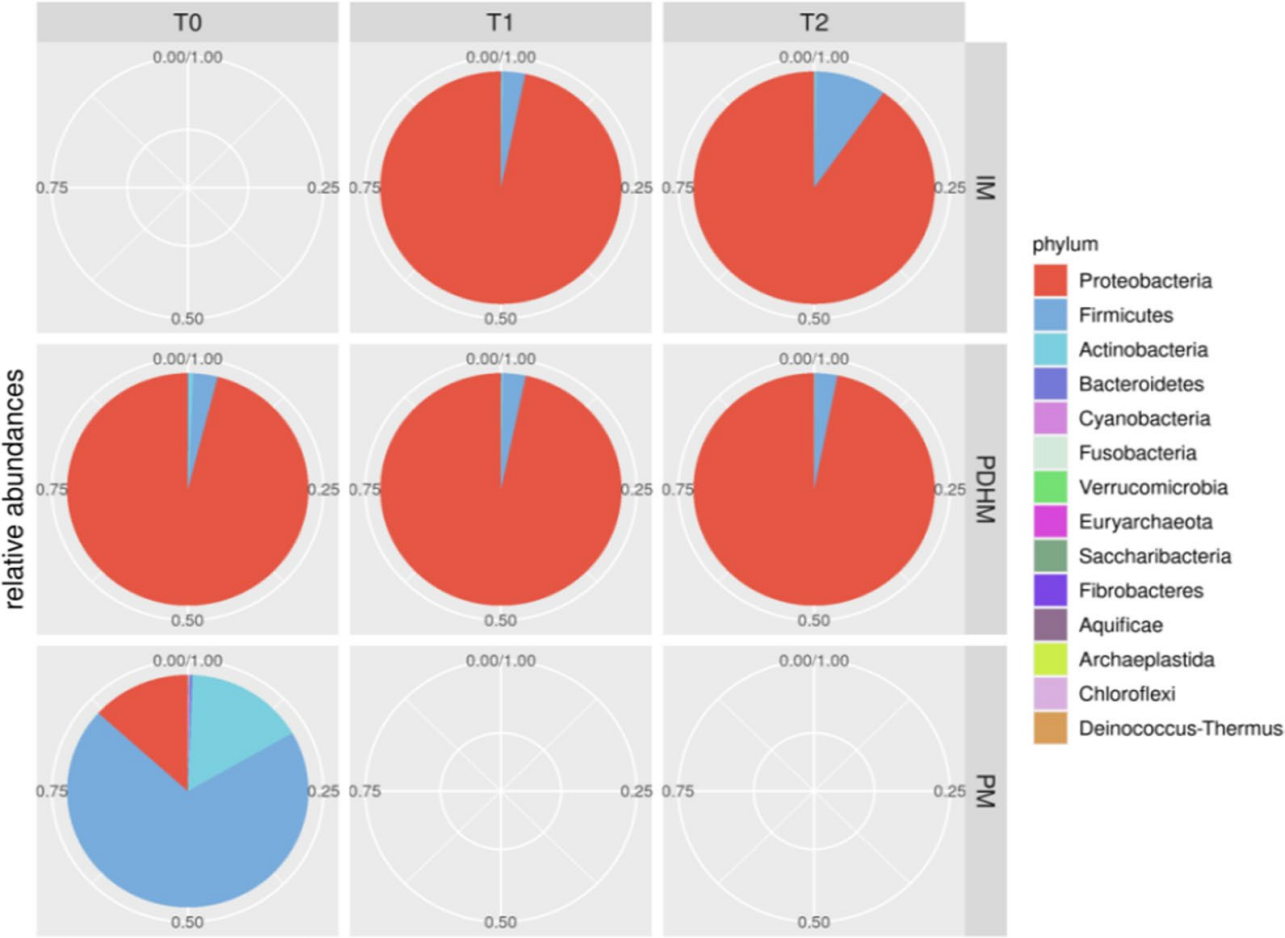
Pie-charts of phylum relative abundances in the PDHM, PM and IM samples. Pie charts showing the distribution of the dominant bacterial phyla in the PDHM, PM and IM samples. The numbers around the pie-charts indicate the percentage of abundance. *PM* preterm milk samples, *PDHM* pasteurized donor human milk samples, *IM* inoculated milk samples. T0: baseline (before inoculum); T1: 2 h after inoculation; T2: 4 h after inoculation

Results from 16S rRNA-gene sequencing from all samples have been used to characterize the core microbiota in PDHM, PM and IM samples. OTUs were grouped taxonomically at the phylum level. In terms of relative abundances, in PM samples, Firmicutes (70%), followed by Actinobacteria (16.1%) and Proteobacteria (13.3%) were the predominant phyla. Instead, most of the reads for PDHM and IM samples belonged to the Proteobacteria and Firmicutes phyla (Fig. 3), which accounted for almost 100% of the entire microbiota at all time points (Table 3). The metataxonomic analysis showed a different microbiota composition for the PDHM and IM samples versus the PM samples. Differences between PDHM and PM samples at T0 were significant for Actinobacteria (p-value = 0.04), Firmicutes (p-value = 0.04) and Proteobacteria (p-value = 0.0006). Upon incubation, the microbial composition of PDHM remained mostly unchanged. In IM samples, Proteobacteria became the predominant phylum (ranged between 81–97%), while Firmicutes moved from 3% at T1 to 9.4% at T2 (Table 3). In IM samples, at the latest timepoint (4 h) Bacteroidetes and Cyanobacteria had a relative abundance lower than 1%.

**Table 3:** Average counts (and relative abundance) in PDHM, PM and IM samples per phylum and time point

Phylum	Time point	PDHM	PM	IM
Actinobacteria	T0	1424.7 (0.7%)	8716.2 (16.1%)	
Actinobacteria	T1	429 (0.3%)		443.3 (0.3%)
Actinobacteria	T2	183 (0.1%)		481.2 (0.3%)
Bacteroidetes	T0	88.7 (0%)	176.5 (0.3%)	
Bacteroidetes	T1	38 (0%)		36.3 (0%)
Bacteroidetes	T2	26.3 (0%)		35.9 (0%)
Cyanobacteria	T0	10.3 (0%)	106.2 (0.2%)	
Cyanobacteria	T1	2.7 (0%)		4.8 (0%)
Cyanobacteria	T2	2.3 (0%)		4.2 (0%)
Firmicutes	T0	6707 (3.2%)	37993.2 (70%)	
Firmicutes	T1	4795 (3%)		5051.1 (3%)
Firmicutes	T2	4066.7 (3%)		14746 (9.4%)
Proteobacteria	T0	198125.3 (96%)	7206 (13.3%)	
Proteobacteria	T1	153568.3 (96.7%)		164498.9 (96.7%)
Proteobacteria	T2	133308.7 (96.9%)		141243.9 (90.2%)

A complete list of the bacterial groups at phylum, family and genus level as well as their relative abundances are reported in Additional file 2: Table S1.

**Fig. 4:**
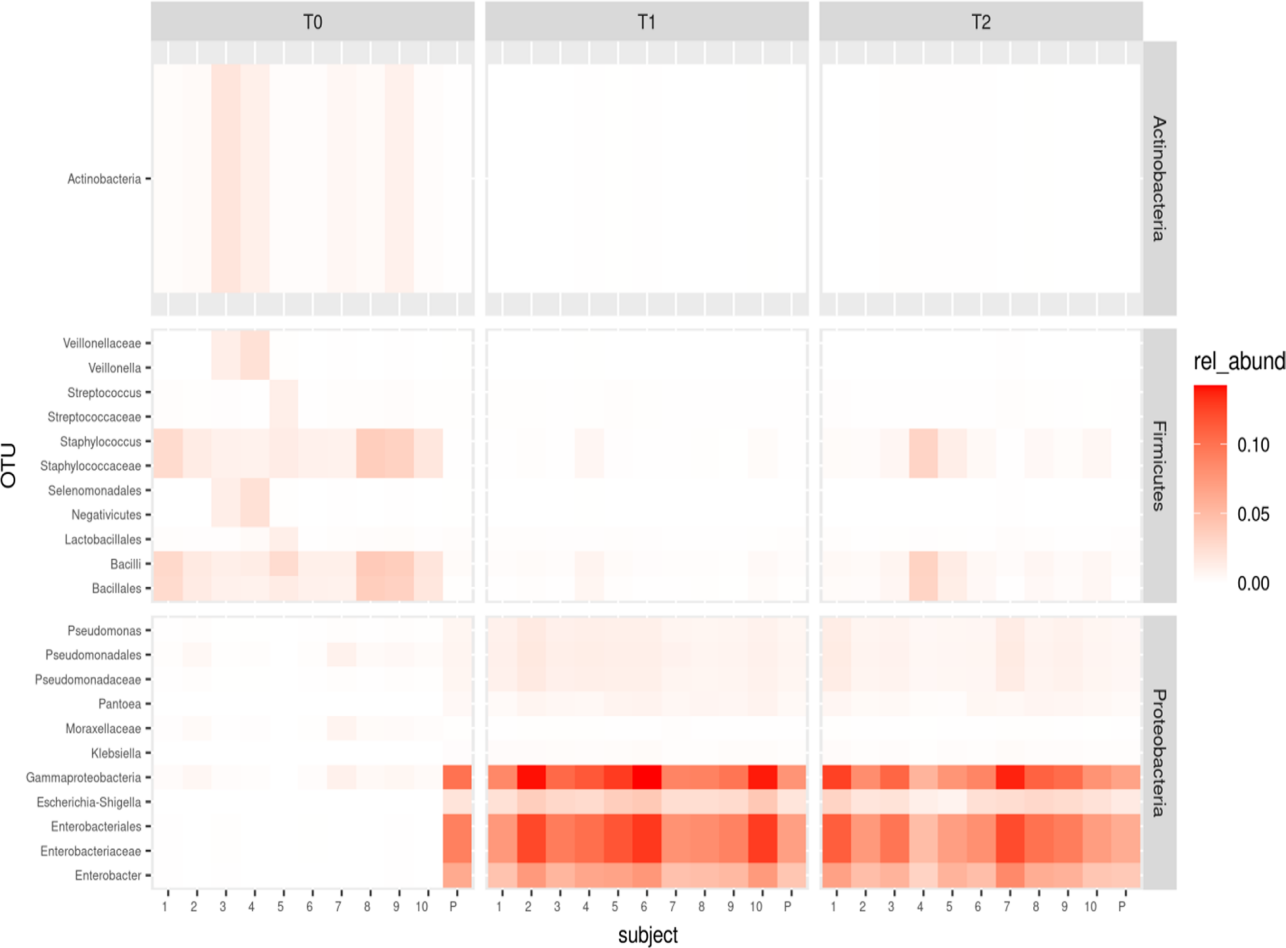
Heatmap of the within-phylum relative abundance of microbial taxa identified in PDHM, PM and IM samples at three time points. The figure shows the relative abundance of microbial taxa belonging to the phylum of Proteobacteria, Firmicutes and Actinobacteria. Sample ‘P’ refers to pasteurized donor human milk before the inoculation which was analyzed at the baseline (T0), 2 h (T1) and 4 h after inoculation (T2). Numbers 1 to 10 in the figure refer to PM1 to PM10 at T0 and IM1 to IM10 at T1and T2. Only taxa with relative abundance larger than 0.25% were considered

These data were further investigated in terms of the relative abundance in bacterial distribution, analyzing each PM sample and IM at T1 and T2 (Fig. 3). Differences in microbial composition between PDHM and PM samples found in the core microbiota analysis are shown in Fig. 4.

At the family level, the microbiota of the PM samples was characterized by *Staphylococcaceae*, with the prevalence of the genus *Staphylococcus*, and Bacillales with the prevalence of the genus *Bacillus*. Lactobacillales family members were observed in few PM samples, with low relative abundance. In PDHM sample, the class of Gammaproteobacteria and the *Enterobacteriaceae* family were the most predominant with Enterobacterales, Enterobacter and *Escherichia-Shigella* genus.

### Variation of several alpha and beta-diversity indexes at different time points of incubation

The alpha-diversity indexes were all significantly moving over time (Table 4). Between-group (PM vs PDHM; IM vs PDHM) differences were significant for the two evenness indexes (equitability and Simpson’s E), while the interaction between groups and time points was significant for the richness (Chao1, ACE, observed OTUs) and diversity (Fisher’s alpha, Shannon) indexes.

**Table 4:** Significance of differences between groups, time points and group-by-time point interactions for the measured alpha-diversity indexes

METRIC	GROUP	TIMEPOINTS	GROUP:TIMEPOINT
Chao1	0.319	0.00017	0.026
ACE	0.282	0.00015	0.021
Fisher’s alpha	0.394	5.4750E−05	0.012
Observed OTUs	0.467	0.00014	0.007
Shannon	0.530	0.00024	0.007
Equitability	0.007	5.51219E−05	0.800
Simpson’s E	0.003	4.41545E−08	0.048

Data reported the p-values from a linear model with the effects of treatment on groups (PM, PDHM, IM), timepoints (T0-T2) and their interaction for each alpha diversity index.

**Fig. 5:**
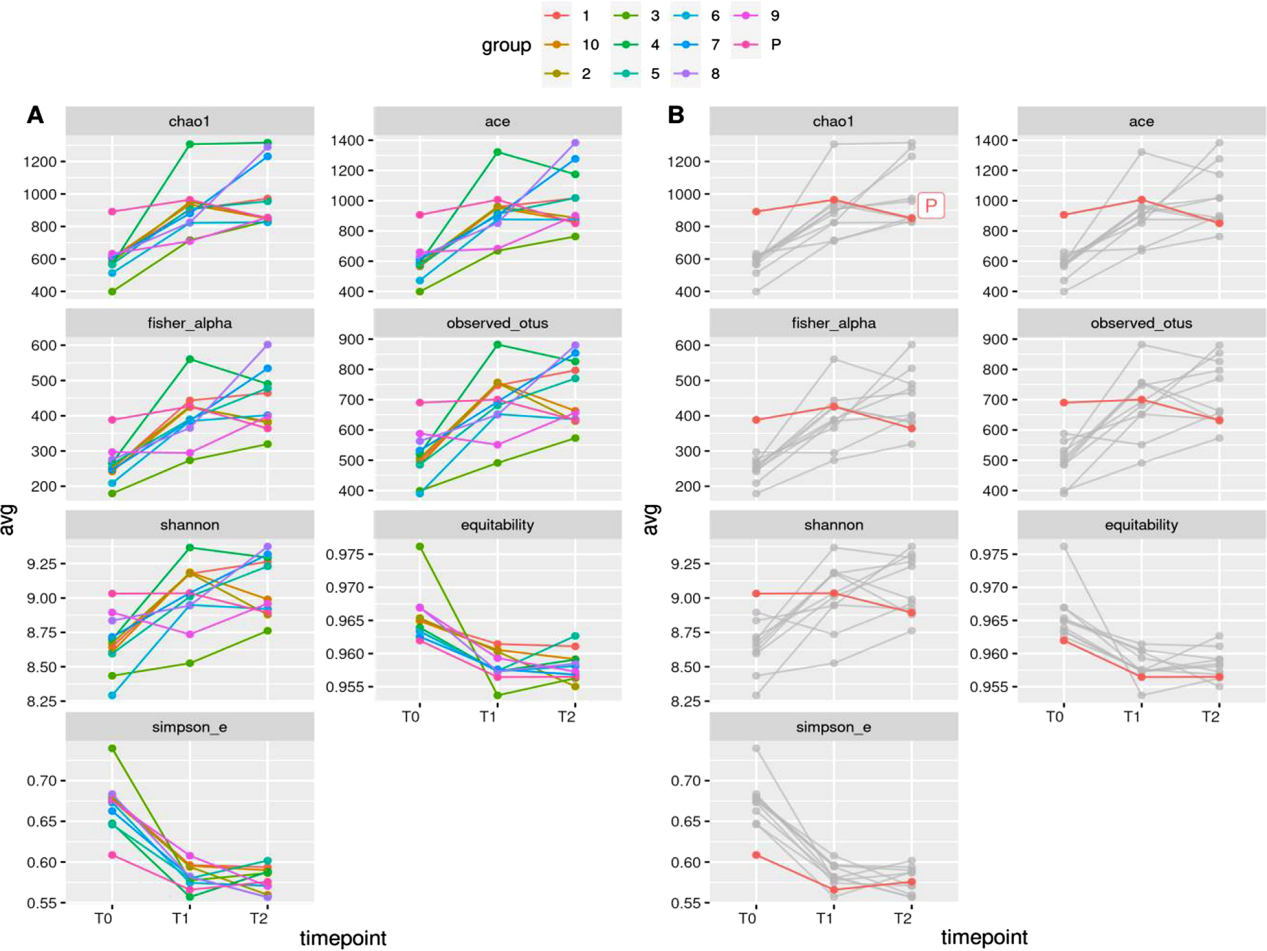
Alpha-diversity analyzed using different indexes per time points. In the **A** alpha-diversity indexes of each sample over time points was reported. In the **B**, the pasteurized donor human milk sample (sample P) has been highlighted. T0: baseline (before inoculum); T1: 2 h after inoculation; T2: 4 h after inoculation

Figure 5 shows the different alpha diversity indexes among samples and over time. For richness and diversity the highest values were consistently found for PDHM at T0: chao1 (891.357 compared to 569.463 of the PM samples), ACE (906.772 vs 567.161), Fisher’s alpha (388.348 vs 248.361), observed-OTUs (690.333 vs 4.966) and Shannon metrics (9.032 vs 8.639). For PDHM samples (highlighted in the right panel of Fig. 5), the alpha diversity was similar along the entire experimental period with values of 886.232, 905.531, 380.92, 628.333 and 8.855 at T2 for chao1, ace, fisher-alpha, observed OTUs and Shannon metrics, respectively.

On the contrary, for most IM samples there was an increment in microbial diversity four hours after the milk incubation at 37 °C (T2), as reported by chao1 (997,815 vs 569,463), ACE (1016,157 vs 567,161), Fisher’s alpha (445,411 vs 248,361) and Shannon (9099 vs 8639) metrics.

**Fig. 6:**
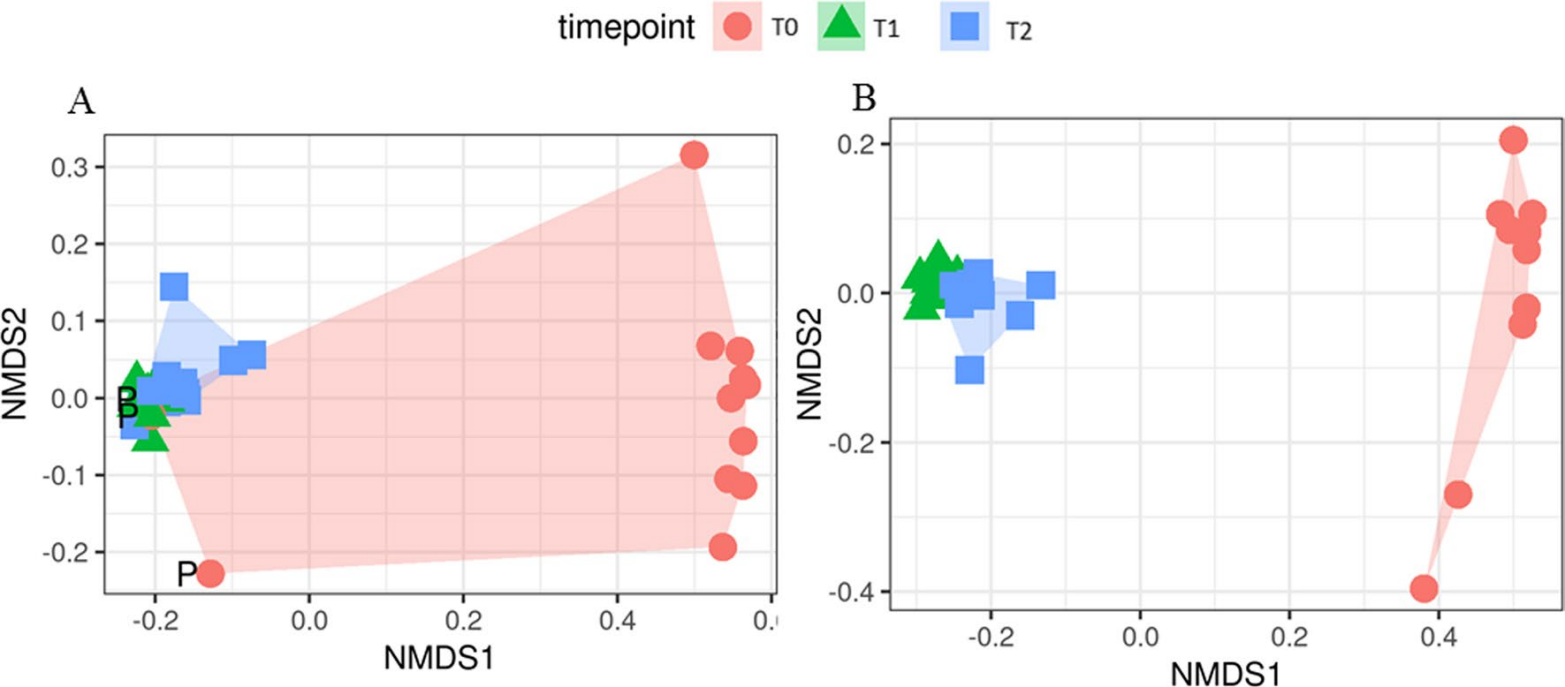
First two dimensions from the (non-metric) multi-dimensional scaling of the Bray–Curtis dissimilarity matrix. Multidimensional scaling (MDS) plot of PM and IM samples in comparison with (Plot **A**) and without (Plot **B**) the PDHM sample. In the plot **A**, the pasteurized donor human milk sample (sample P) has been highlighted. Samples were grouped by each time point (T0, T1, T2) shown by a shape (circle, triangle and square, respectively)

Beta-diversity analysis was evaluated with (Fig. 6A) and without (Fig. 6B) the PDHM sample. From PERMANOVA (999 permutations), the analysis showed a statistically significant (p = 0.001) separation between timepoints and hence between PM and IM samples, revealing major differences in the principal constituents of the microbial community (Fig. 6B). PDHM presented a different microbial community as compared to the PM (Fig. 6A) samples, and, being the substrate for the inoculum, it determined microbial composition of IM samples within 4 h of incubation.

### Peptidomic analysis of PDHM, PM and IM samples

**Fig. 7:**
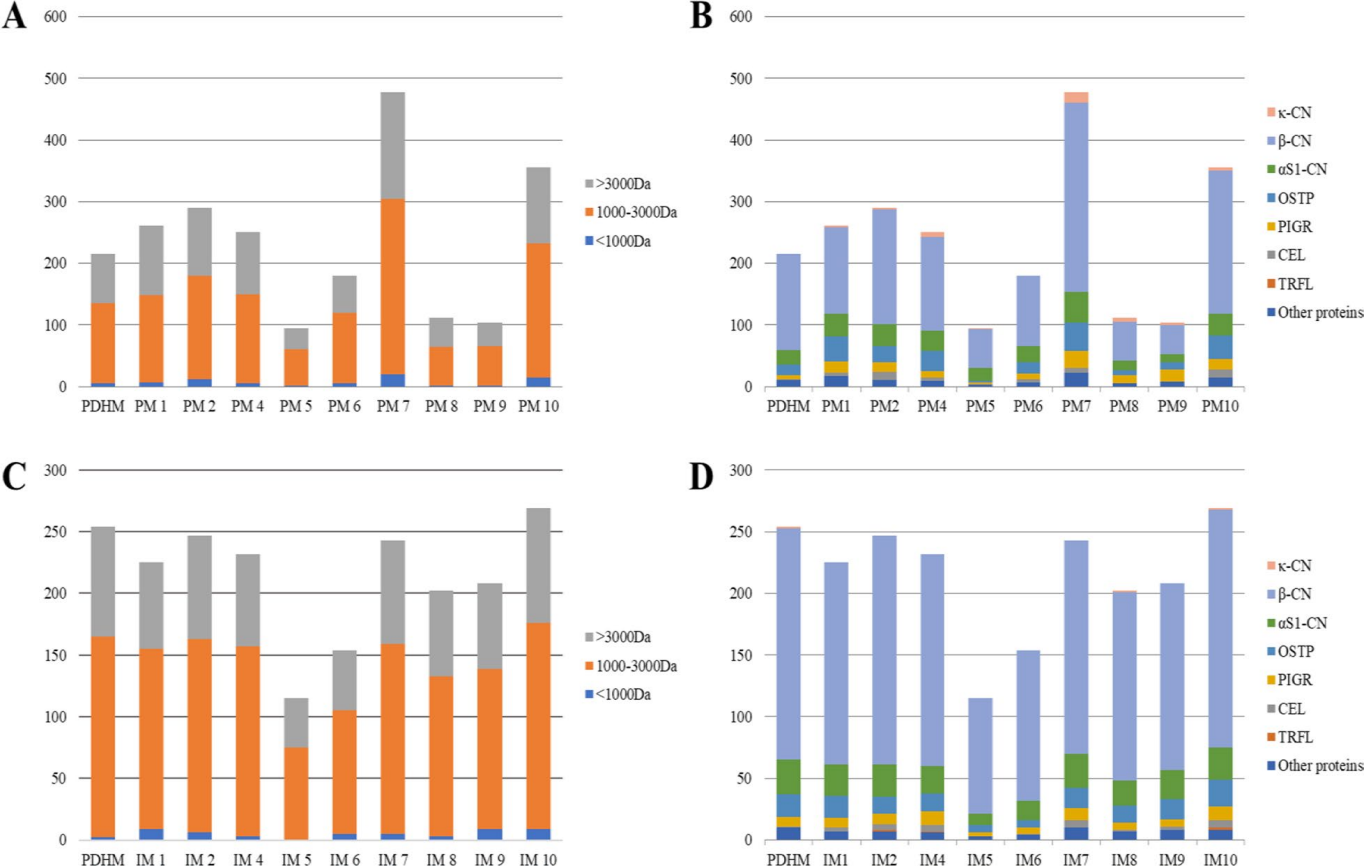
Qualitative peptidomic overview of PDHM, PM and IM samples. Number and molecular weight distribution of the peptides identified in PDHM and PM at T0 (**A**) and PDHM and IM at T2 (**C**). Number of peptides and related precursors proteins in PDHM and PM at T0 (**B**) and PDHM and IM at T2 (**D**). (κ-CN, κ-casein; β-CN, β-casein; αS1-CN, αS1-casein; OSTP, osteopontin; PIGR, polymeric immunoglobulin receptor; CEL, bile salt-activated lipase; TRFL, lactotransferrin)

A qualitative peptidomic analysis was conducted on PDHM, PM and IM samples at different time points. Since PM3 and PM5 were collected from the same mother and their peptidomic profiles overlapped, only the PM5 was considered for the discussion. In PM samples, about 800 unique peptides were identified. Each PM contained a different number of peptides, higher (PM1, PM2, PM4, PM7, PM10) or lower (PM5, PM6, PM8, PM9) than PDHM. As shown in Fig. 7, PM7 and PM10 appeared as the most proteolyzed samples, whereas the lowest extent of proteolysis characterized PM 5, 8 and 9. At T0 about 60% of total peptides identified both in PDHM and PM samples were characterized by a molecular weight ranging between 1 and 3 kDa, while those smaller than 1 kDa represented only 3% of total peptides (Fig. 7A). Human β-casein was the most representative protein, with 62% of total peptides, followed by α_S1_-casein (13%), osteopontin (10%) and polymeric immunoglobulin receptor (6%) (Fig. 7B). Peptides belonging to α-lactalbumin, one of the most abundant proteins in HM, were not found. At T2, the number and type of peptides in PDHM sample roughly overlapped those revealed at T0. As a matter of fact, incubation for 4 h at 37 °C did not alter the peptidomic profiles of PDHM sample. The peptidomic profile of IM samples, except for IM5 and IM6, resembled that of PDHM incubated for the same time (Fig. 7C and Fig. 7D). The pattern of precursor proteins identified in IM samples at the end of incubation (T2) was a reflection of PDHM’s one (Fig. 7D). The dominance of β-casein derived peptides (74% of total peptides identified at T2) was confirmed in all IM samples. Peptides in the molecular weight range of 1–3 kDa were still the most abundant (Fig. 7C).

## Discussion

In this study, a personalization of PDHM in terms of bacterial growth and human milk microbiome emerged after inoculation with mother’s own milk. The present study is one of the few investigations on microbiome and peptidome of PDHM, PM and derived IM samples, and it can be considered the first one dealing with the occurrence and potential growth of pathogenic bacteria in PM and related incubated IM samples.

Several studies [49, 50] showed that HM is not sterile and contains a variety of mother-specific probiotic and commensal bacteria, which constitute HM microbiome, a source of potential beneficial bacteria for the colonization of preterm infants’ gut.

The origin of bacteria in HM depends on different factors. Currently it is possible to distinguish between a “maternal microbiota” and an “exogenous microbiota”, as supported by Moossavi & Azad [51]. In addition to the transfer of microbes from breast skin flora to the milk through its expression, there are other pathways relevant in the shaping of a specific HM microbiome such as contamination related to milk handling.

A recent review of Fernández & Rodríguez confirmed that a bacterial load of HM from healthy women, collected in appropriate hygienic condition, usually was about 3 log_10_ CFU/mL while a bacterial load up to 6 log_10_ CFU/mL was characteristic of maternal mastitis infection or milk contamination after pumping [52]. The bacterial load of our PM samples ranged from 3 to 5 log_10_ CFU/mL: nevertheless the strict adherence to hygienic condition in Neonatal Intensive Care Unit, an external contamination of expressed HM during breast milk collection process can’t be excluded. Our results show that the inoculation of PDHM with PM (10% v/v) followed by 4 h of incubation at 37 °C allowed the restoration of bacterial load similar to that of PM, although an high variability of bacterial growth between PM samples and consequently in IM samples, was detected.

At T2 all microorganisms grown in PM samples were found in the respective IM samples.

Specifically, in this study, cultivable bacteria as LAB or belonging to *Staphylococcaceae, Streptococcaceae*, *Enterobacteriaceae* and *Pseudomonaceae* and other gram-positive bacteria (*Propionibacterium* and *Bifidobacterium*) were detected in PM samples and in their related IM samples.

Most of the species belonging to the genera *Lactobacillus* and *Bifidobacterium*, commonly considered probiotic bacteria, can confer health benefits to HM-fed premature infants. LAB were found in all PM samples, while they were not found in PDHM even if they reached the same load in all IM samples at the end of incubation period. Some species of lactobacilli, commonly present in human breast milk, colonize the neonatal gut and contribute to protection against infant infections, thus suggesting that lactobacilli could be potentially beneficial in the modulation of immunity [53]. HM lactobacilli strains are metabolically active in the infant gut and increase the production of functional metabolites such as butyrate, which is not only the main energy source for colonocytes but also a relevant compound in the modulation of intestinal function [54]. In addition, a bacteriostatic and bactericidal effect of some Lactobacillus species, on pathogenic microorganisms, such as Staphilococcus aureus and Salmonella enterica, have been demonstrated *in-vitro* and in a mouse model [4].

Concerning Bifidobacteria, PM contains lower levels compared to milk of term-delivering mothers [55]. Moreover, indirect breastfeeding could affect the concentration of Bifidobacteria in HM, which can be further lowered by the need to use breast-pump [2]. In addition, it should be considered that Bifidobacteria grow only in strict anaerobic condition. This could explain our difficulty to detect Bifidobacteria in PM and IM samples using traditional plate count method, as confirmed also by Boix-Amorós et al. [56].

The presence in PM samples of microbial families belonging to *Staphylococcaceae, Streptococcaceae, Enterobacteriaceae* and *Pseudomonaceae* could be consistent with contamination during breast milk collection, even if hygienic protocols are followed [2, 57, 58].

Given that pasteurization, prior the administration, of mother’s own milk is not recommended [59], also potential pathogenic microorganisms are normally conveyed to preterm infants fed with fresh HM. Nevertheless, breastfeeding is recommended because the potential benefits of breast milk outweigh the possible negative effects: only few articles reported that neonatal infection was caused by the ingestion of contaminated HM [60].

The feeding with HM to the newborn should be avoided or stopped only in specific circumstances [61]. Particular attention should be paid to the implementation of the hygienic measures during milk collection, in the Neonatal Intensive Care Unit and at home, which could avoid proliferation of undesirable bacteria in expressed HM.

DHM must be necessarily subjected to Holder pasteurization treatment, in order to neutralize the potential pathogenic bacterial load present. Such treatment unfortunately destroy also beneficial bacteria, such as LAB and bifidobacteria [62]. In our study, metataxonomic analysis underlined that the addition of a small amount of PM can partially restore the microbiome of PDHM, making it more similar to those of PM.

In accordance with other studies [3, 63], the microbiota of our PM samples was mostly characterized by the presence of *Staphylococcaceae*, with a prevalence of the *Staphylococcus* genus, and Bacillales with a prevalence of the *Bacillus* genus. The large number of Firmicutes in these samples could be due to the physiological presence of bacteria belonging to this phylum on areolar skin.

Conversely, microbiota of our PDHM sample was mostly characterized by Proteobacteria, which is the dominant phylum in HM as also confirmed by different studies [63, 64].

Inoculation and incubation determined an enrichment of microbiota of IM in terms of Firmicutes, which represent predominant phylum of our PM samples. The presence of *Staphylococcaceae* and Bacillales, found in IM samples at T2, was representative of the microbial enrichment due to inoculum leading to a microbiota profile more similar to those of PM. In addition, an overall increase of microbial diversity in IM samples at four hours after the incubation was detected. Given that PDHM’s contribution in terms of inoculum volumes was predominant, we hypothesized that the microbial diversity of IM samples could be a reflection of those of PDHM, whose microbial diversity indexes at T0 were not only higher than those of PM, but they also remained unaltered during the entire period of incubation. Higher microbial diversity of PDHM found in our study was in accordance with Garcia-Gonzalez et al. [64]. They hypothesized that pasteurization process could select thermoduric bacteria, capable to survive the thermic process, thus increasing their abundance. Furthermore, given that alpha diversity indexes of IM samples at the end of incubation were higher than those of PDHM, we argue that this higher values could be principally attributable to PM used for inoculation. In fact, the incubation seems to be not effective on microbial diversity of PDHM.

Recent findings [65] suggest that bacteria in breast milk may be transferred to the infants influencing the development of their gut microbiota. Younge et al. found that the microbiota of preterm infants, with postnatal growth failure, had a constantly low alpha diversity compared to preterm infants with an appropriate growth [66]. The increase of PDHM’s microbial diversity after inoculation, could avoid dysbiosis, allowing a balanced maturation of the intestinal microbiota.

Studies on the impact of different nutritional approaches on the preterm infants’ gut microbiota are very limited and not always in accordance each other.

Ford et al. demonstrated that preterm infants fed with mother’s own milk developed an increased gut microbial community compared to preterm infants fed with PDHM: they also had a better weight gain and an improved feeding tolerance than PDHM-fed infants [68]. On the other hands, many studies [6, 67–69] suggest that PDHM favors an intestinal microbial profile more similar to those of mother’s own milk.

Anyway, the inoculation strategy could lead to a personalization of PDHM which could shape infants’ gut microbiota increasing microbial diversity and promoting healthier short and long-term outcomes in preterm infants. Increasing volumes of mother’s own milk for inoculation could be more useful: higher volumes in fact, could result in higher microbial diversity of IM samples. However it should be considered microbiological quality of mother’s own milk which has to be used for inoculation: a pathogenic flora, if presents, could be amplified with inoculation and incubation potentially leading to adverse effect on preterm infants.

As described by Fernández et al. [54] HM bacteria also showed a remarkable potential metabolic roles in the infant and it might also contribute to implement infant digestion through the breakdown of sugars and proteins. A recent study about metabolomics profile of donor HM identified several compounds as a result of microbial activity [70]. Despite these evidences, in this study the peptidomic profile of PDHM was slightly affected by incubation. Probably this could be due to the fact that it was previously stored at − 20 °C and then pasteurized. In fact, as demonstrated by Ahrabi et al. [71], freezer storage of donor HM at − 20 °C is associated with a decreasing bacterial count. It is known that the Holder pasteurization process not only reduced the bacterial count, but it also inactivated most of the bacterial and endogenous proteolytic enzymes [72, 73].

Each PM sample presented different degree of proteolysis that was reflected in specific peptidomic profiles. Despite the inter-variability of PM samples, negligible differences in proteolysis level and peptidomic profile among IM samples were detected over time. Overall, these features mostly reflected those of PDHM sample at the same time of incubation, thus underlying a negligible exogenous proteolysis due to the activity of microbial communities restored after inoculum. The largest number of peptides in PDHM and PM samples derived from β-casein and, therefore, the same appeared for IM samples. These finding is in accordance with those of different authors who demonstrated the prevalence of β-casein derived peptides in HM [74–76]. Differently, a scarce presence of peptides derived from major whey proteins, in particular from α-lactalbumin, was found in our studied samples, as also reported for HM by Gan et al. [77]. In summary, inoculation strategy could be beneficial being able to restore mother’s own milk microbial community in PDHM and personalizing its microbiome. On the other hand, the peptidomic profile seems to be not affected by inoculation strategy. Overall, the feasibility of inoculating PDHM with fresh PM for preterm infant feeding, needs further investigation: in-vitro studies, taking into account not only a larger sample size but also increasing percentage of inoculated volume (% v/v), should be conducted. Prior to translate inoculation strategy in the clinical practice, *in-vivo* studies and further insights are required to evaluate not only the safety but also its feasibility.

## Conclusions

The present research study demonstrated that inoculation with mother’s own milk restores microbial community and personalizes HM microbiome of PDHM. This effect could be beneficial for preterm infants, given the presence of maternal probiotic bacteria which make inoculated PDHM more similar to mother’s own milk. The unique fingerprint-like microbiota of PM samples used for inoculation allows the personalization of inoculated PDHM, thus possibly contributing to enhance the bacterial diversity of infant’s gut microbiota. The feasibility of inoculation strategy should be reinforced by further studies.

## Supplementary Information

**Additional file 1: Figure S1.** Number of OTUs plotted as function of number of samples’ reads and number of samples.

**Additional file 2: Table S1.** A complete list of the bacterial groups at phylum, family and genus and their relative abundances.

## Data Availability

The authors confirm that the data supporting the findings of this study are available within the article upon reasonable request.

## Abbreviations

HM: Human milk
PDHM: Pasteurized donor human milk
PM: Preterm mother’s own milk
IM: Inoculated milk (PDHM inoculated with PM)
NICU: Neonatal Intensive Care Unit
LAB: Lactic acid bacteria

## References

[1] Cheng L, Akkerman R, Kong C, Walvoort MTC, de Vos P (2020). More than sugar in the milk: human milk oligosaccharides as essential bioactive molecules in breast milk and current insight in beneficial effects. Crit Rev Food Sci Nutr.

[2] Moossavi S, Sepehri S, Robertson B, Bode L, Goruk S, Field CJ (2019). Composition and variation of the human milk microbiota are influenced by maternal and early-life factors. Cell Host Microbe.

[3] Gomez-Gallego C, Garcia-Mantrana I, Salminen S, Collado MC (2016). The human milk microbiome and factors influencing its composition and activity. Semin Fetal Neonatal Med.

[4] Selma-Royo M, Calvo Lerma J, Cortés-Macías E, Collado MC (2021). Human milk microbiome: from actual knowledge to future perspective. Semin Perinatol.

[5] Cacho NT, Harrison NA, Parker LA, Padgett KA, Lemas DJ, Marcial GE (2017). Personalization of the microbiota of donor human milk with mother’s own milk. Front Microbiol.

[6] Cong X, Genomics S, Judge M, Xu W, Diallo A, Janton S (2017). Influence of infant feeding type on gut microbiome development in hospitalized preterm infants. Nurs Res.

[7] Wilson E, Edstedt Bonamy AK, Bonet M, Toome L, Rodrigues C, Howell EA (2018). Room for improvement in breast milk feeding after very preterm birth in Europe: results from the EPICE cohort. Matern Child Nutr.

[8] Eidelman AI, Schanler RJ (2012). Breastfeeding and the use of human milk. Pediatrics.

[9] Human Milk Banking Association of North America. Best practice for expressing, storing and handling human milk in hospitals, homes, and child care settings. HMBANA. 4th ed. 2019. https://www.hmbana.org/our-work/publications.html.

[10] Weaver G, Bertino E, Gebauer C, Grovslien A, Mileusnic-Milenovic R, Arslanoglu S (2019). Recommendations for the establishment and operation of Human Milk Banks in Europe: a consensus statement from the European Milk Bank Association (EMBA). Front Pediatr.

[11] Cacho NT, Lawrence RM (2017). Innate immunity and breast milk. Front Immunol.

[12] Beghetti I, Biagi E, Martini S, Brigidi P, Corvaglia L, Aceti A (2019). Human milk’s hidden gift: implications of the milk microbiome for preterm infants’ health. Nutrients.

[13] Zhu J, Dingess KA (2019). The functional power of the human milk proteome. Nutrients.

[14] Arslanoglu S, Bertino E, Tonetto P, De Nisi G, Ambruzzi AM, Biasini A (2010). Guidelines for the establishment and operation of a donor human milk bank. J Matern Neonatal Med.

[15] ISO 21527-1:2008. Microbiology of food and animal feeding stuffs—horizontal method for the enumeration of yeasts and moulds Colony count technique in products with water activity greater than 0,95.

[16] Zucali M, Bava L, Colombini S, Brasca M, Decimo M, Morandi S (2015). Management practices and forage quality affecting the contamination of milk with anaerobic spore-forming bacteria. J Sci Food Agric.

[17] ISO 29981:2010. Milk products—enumeration of presumptive bifidobacteria—colony count technique at 37 degrees C.

[18] ISO/TS 11059:2009. Milk and milk products—method for the enumeration of *Pseudomonas* spp.

[19] ISO 21871:2006. Microbiology of food and animal feeding stuffs—horizontal method for the determination of low numbers of presumptive *Bacillus cereus*—most probable number technique and detection method.

[20] ISO/NP 6888–3:2003. Microbiology of food and animal feeding stuffs—horizontal method for the enumeration of coagulase-positive staphylococci (*Staphylococcus aureus* and other species)—part 3: detection and MPN technique for low numbers.

[21] Cremonesi P, Pisani LF, Lecchi C, Ceciliani F, Martino P, Bonastre AS (2014). Development of 23 individual TaqMan^®^ real-time PCR assays for identifying common foodborne pathogens using a single set of amplification conditions. Food Microbiol.

[22] Cremonesi P, Perez G, Pisoni G, Moroni P, Morandi S, Luzzana M (2007). Detection of enterotoxigenic *Staphylococcus aureus* isolates in raw milk cheese. Lett Appl Microbiol.

[23] Cremonesi P, Luzzana M, Brasca M, Morandi S, Lodi R, Vimercati C (2005). Development of a multiplex PCR assay for the identification of *Staphylococcus aureus* enterotoxigenic strains isolated from milk and dairy products. Mol Cell Probes.

[24] Cremonesi P, Ceccarani C, Curone G, Severgnini M, Pollera C, Bronzo V (2018). Milk microbiome diversity and bacterial group prevalence in a comparison between healthy holstein friesian and rendena cows. PLoS ONE.

[25] Caporaso JG, Lauber CL, Walters WA, Berg-Lyons D, Lozupone CA, Turnbaugh PJ (2011). Global patterns of 16S rRNA diversity at a depth of millions of sequences per sample. Proc Natl Acad Sci USA.

[26] Andrews S. FastQC: a quality control tool for high throughput sequence data. 2010. http://www.bioinformatics.babraham.ac.uk/projects/fastqc/. Accessed 04 2021.

[27] John JA. SeqPrep v1.1. Tool for stripping adaptors and/or merging paired reads with overlap into single reads. 2011. https://github.com/jstjohn/SeqPrep. Accessed 04 2021.

[28] Quast C, Pruesse E, Yilmaz P, Gerken J, Schweer T, Yarza P (2013). The SILVA ribosomal RNA gene database project: improved data processing and web-based tools. Nucleic Acids Res.

[29] Yilmaz P, Parfrey LW, Yarza P, Gerken J, Pruesse E, Quast C (2014). The SILVA and “all-species living tree project (LTP)” taxonomic frameworks. Nucleic Acids Res.

[30] Weizhong L, Godzik A (2006). Cd-hit: a fast program for clustering and comparing large sets of protein or nucleotide sequences. Bioinformatics.

[31] Caporaso JG, Kuczynski J, Stombaugh J, Bittinger K, Bushman FD, Costello EK (2010). QIIME allowsanalysis of high-throughput community sequencing data. Nature.

[32] Chao A (1984). Nonparametric estimation of the number of classes in a population author. Scan J Stat.

[33] Chao A, Lee SM (1992). Estimating the number of classes via sample coverage. J Am Stat Assoc.

[34] Chao A, Ma MC, Yang MCK (1993). Stopping rule and estimation for recapture debugging with unequal detection rates. Biometrika.

[35] Shannon C (1948). A mathematical theory of communication, the bell system technical journal. Bell Syst Techn J.

[36] Simpson EH (1949). Measurement of diversity. Nature.

[37] Fisher RA, Corbet AS, Williams CB (1943). The relation between the number of species and the number of individuals in a random sample of an animal population. J Anim Ecol.

[38] Smith B, Wilson JB (1996). A consumer’s guide to evenness indices. Oikos.

[39] Bray JR, Curtis JT (1957). An ordination of the upland forest communities of southern Wisconsin. Ecol Monogr.

[40] Paulson JN, Colin Stine O, Bravo HC, Pop M (2013). Differential abundance analysis for microbial marker-gene surveys. Nat Methods.

[41] Anderson M (2001). A new method for non-parametric multivariate analysis of variance. Austr Ecol.

[42] Biscarini F, Palazzo F, Castellani F, Masetti G, Grotta L, Cichelli A (2018). Rumen microbiome in dairy calves fed copper and grape-pomace dietary supplementations: composition and predicted functional profile. PLoS ONE.

[43] Mangé A, Bellet V, Tuaillon E, Van de Perre P, Solassol J (2008). Comprehensive proteomic analysis of the human milk proteome: contribution of protein fractionation. J Chromatogr B Anal Technol Biomed Life Sci.

[44] Tabb DL (2015). The SEQUEST family tree. J Am Soc Mass Spectrom.

[45] Pica V, Stuknytė M, Masotti F, De Noni I, Cattaneo S (2021). Bovine milk fortifiers and fortified pasteurized donor human milk for premature infant nutrition. Peptidomic overview. LWT.

[46] Liao Y, Alvarado R, Phinney B, Lönnerdal B (2011). Proteomic characterization of human milk fat globule membrane proteins during a 12 month lactation period. J Proteome Res.

[47] Lönnerdal B (2003). Nutritional and physiologic significance of human milk proteins. Am J Clin Nutr.

[48] Molinari CE, Casadio YS, Hartmann BT, Livk A, Bringans S, Arthur PG (2012). Proteome mapping of human skim milk proteins in term and preterm milk. J Proteome Res.

[49] Lyons KE, Ryan CA, Dempsey EM, Ross RP, Stanton C (2020). Breast milk, a source of beneficial microbes and associated benefits for infant health. Nutrients.

[50] Stinson LF, Sindi ASM, Cheema AS, Lai CT, Mühlhäusler BS, Wlodek ME (2020). The human milk microbiome: who, what, when, where, why, and how?. Nutr Rev.

[51] Moossavi S, Azad MB (2020). Origins of human milk microbiota: new evidence and arising questions. Gut Microbes.

[52] Fernández L, Rodríguez JM (2020). Human milk microbiota: origin and potential uses. Nestle Nutr Inst Workshop Ser.

[53] Pérez-Cano FJ, Dong H, Yaqoob P (2010). In vitro immunomodulatory activity of *Lactobacillus fermentum* CECT5716 and *Lactobacillus salivarius* CECT5713: two probiotic strains isolated from human breast milk. Immunobiology.

[54] Fernández L, Langa S, Martín V, Maldonado A, Jiménez E, Martín R (2013). The human milk microbiota: origin and potential roles in health and disease. Pharmacol Res.

[55] Khodayar-Pardo P, Mira-Pascual L, Collado MC, Martínez-Costa C (2014). Impact of lactation stage, gestational age and mode of delivery on breast milk microbiota. J Perinatol.

[56] Boix-Amorós A, Collado MC, Mira A (2016). Relationship between milk microbiota, bacterial load, macronutrients, and human cells during lactation. Front Microbiol.

[57] Jiménez E, Arroyo R, Cárdenas N, Marín M, Serrano P, Fernández L (2017). Mammary candidiasis: a medical condition without scientific evidence?. PLoS ONE.

[58] Sakwinska O, Moine D, Delley M, Combremont S, Rezzonico E, Descombes P (2016). Microbiota in breast milk of Chinese lactating mothers. PLoS ONE.

[59] Vincent M, Ménard O, Etienne J, Ossemond J, Durand A, Buffin R (2020). Human milk pasteurisation reduces pre-lipolysis but not digestive lipolysis and moderately decreases intestinal lipid uptake in a combination of preterm infant in vitro models. Food Chem.

[60] Widger J, O’Connell NH, Stack T (2010). Breast milk causing neonatal sepsis and death. Clin Microbiol Infect.

[61] Civardi E, Garofoli F, Tzialla C, Paolillo P, Bollani L, Stronati M (2013). Microorganisms in human milk: lights and shadows. J Matern Neonatal Med.

[62] Fernández L, Ruiz L, Jara J, Orgaz B, Rodríguez JM (2018). Strategies for the preservation, restoration and modulation of the human milk microbiota. Implications for human milk banks and neonatal intensive care units. Front Microbiol.

[63] Pannaraj PS, Li F, Cerini C, Bender JM, Yang S, Rollie A (2017). Association between breast milk bacterial communities and establishment and development of the infant gut microbiome. JAMA Pediatr.

[64] García-González I, Corona-Cervantes K, Hernández-Quiroz F, Villalobos-Flores LE, Galván-Rodríguez F, Romano MC (2021). The effect of holder pasteurization on the diversity of the human milk bacterial microbiota using high-throughput DNA sequencing. J Hum Lact.

[65] Fehr K, Moossavi S, Sbihi H, Boutin RCT, Bode L, Robertson B (2020). Breastmilk feeding practices are associated with the co-occurrence of bacteria in mothers’ milk and the infant gut: the CHILD cohort study. Cell Host Microbe.

[66] Younge NE, Newgard CB, Cotten CM, Goldberg RN, Muehlbauer MJ, Bain JR (2019). Disrupted maturation of the microbiota and metabolome among extremely preterm infants with postnatal growth failure. Sci Rep.

[67] Parra-Llorca A, Gormaz M, Alcántara C, Cernada M, Nuñez-Ramiro A, Vento M (2018). Preterm gut microbiome depending on feeding type: significance of donor human milk. Front Microbiol.

[68] Ford SL, Lohmann P, Preidis GA, Gordon PS, O’Donnell A, Hagan J (2019). Improved feeding tolerance and growth are linked to increased gut microbial community diversity in very-low-birth-weight infants fed mother’s own milk compared with donor breast milk. Am J Clin Nutr.

[69] Morais J, Marques C, Teixeira D, Durão C, Faria A, Brito S (2019). FEEDMI: a study protocol to determine the influence of infant-feeding on very-preterm-infant’s gut microbiota. Neonatology.

[70] Torrez Lamberti MF, DeBose-Scarlett E, Garret T, Parker LA, Neu J, Lorca GL (2020). Metabolomic profile of personalized donor human milk. Molecules.

[71] Ahrabi AF, Handa D, Codipilly CN, Shah S, Williams JE, McGuire MA (2016). Effects of extended freezer storage on the integrity of human milk. J Pediatr.

[72] Wesolowska A, Sinkiewicz-Darol E, Barbarska O, Bernatowicz-Lojko U, Borszewska-Kornacka MK, van Goudoever JB (2019). Innovative techniques of processing human milk to preserve key components. Nutrients.

[73] Paulaviciene IJ, Liubsys A, Eidukaite A, Molyte A, Tamuliene L, Usonis V (2020). The effect of prolonged freezing and holder pasteurization on the macronutrient and bioactive protein compositions of human milk. Breastfeed Med.

[74] Deglaire A, De Oliveira S, Jardin J, Briard-Bion V, Kroell F, Emily M (2019). Impact of human milk pasteurization on the kinetics of peptide release during in vitro dynamic digestion at the preterm newborn stage. Food Chem.

[75] Dallas DC, Guerrero A, Khaldi N, Borghese R, Bhandari A, Underwood MA (2014). A peptidomic analysis of human milk digestion in the infant stomach reveals protein-specific degradation patterns. J Nutr.

[76] Wada Y, Lönnerdal B (2015). Bioactive peptides released from in vitro digestion of human milk with or without pasteurization. Pediatr Res.

[77] Gan J, Robinson RC, Wang J, Krishnakumar N, Manning CJ, Lor Y (2018). Peptidomic profiling of human milk with LC–MS/MS reveals pH-specific proteolysis of milk proteins. Food Chem.

