# Supplementary figures and images for: Inoculation of mother’s own milk could personalize pasteurized donor human milk used for feeding preterm infants
Source: J Transl Med. 2021 Oct 9;19:420. doi: 10.1186/s12967-021-03096-7 (PMC8502300; doi:10.1186/s12967-021-03096-7)

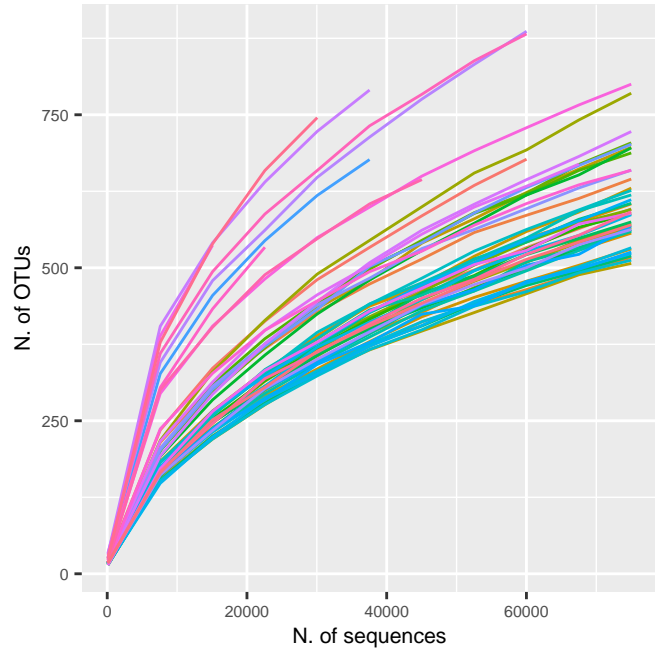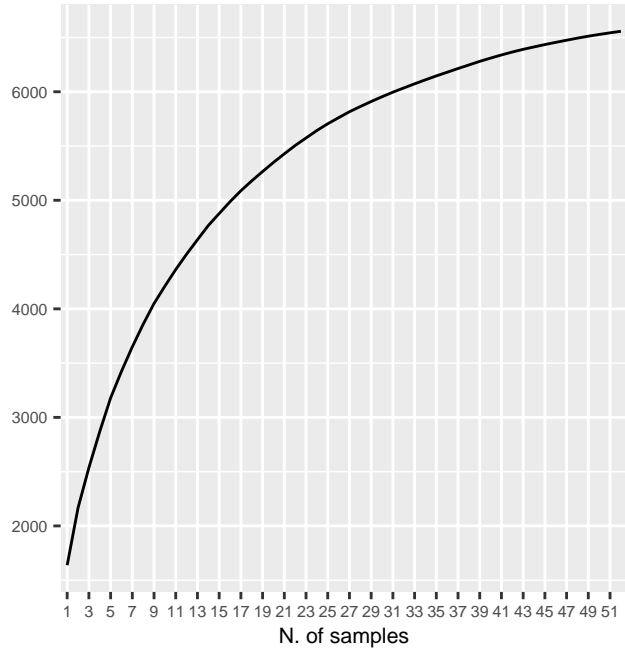

Supplement: Supplementary file 1 — Additional file 1: Figure S1. Number of OTUs plotted as function of number of samples’ reads and number of samples. [file 12967_2021_3096_MOESM1_ESM.pdf]
