# Supplementary material for: Inoculation of mother’s own milk could personalize pasteurized donor human milk used for feeding preterm infants
Source: J Transl Med. 2021 Oct 9;19:420. doi: 10.1186/s12967-021-03096-7 (PMC8502300; doi:10.1186/s12967-021-03096-7)
Supplement: Supplementary file 2 — Additional file 2: Table S1. A complete list of the bacterial groups at phylum, family and genus and their relative abundances. [file 12967_2021_3096_MOESM2_ESM.pdf]

Additional Table 1

A complete list of the bacterial groups at phylum, family and genus and their relative abundances.

level	taxon	group	T0	T1	T2
genus	3	5	0,0004	0	0
genus	3	8	0	0	0,0001
genus	4	6	0,0003	0	0
genus	4	9	0,0024	0	0
genus	5	1	0	0	0,0001
genus	5	5	0,0004	0	0
genus	5	7	0,0076	0	0
genus	5	8	0,0002	0	0
genus	5	9	0,0008	0	0
genus	13	5	0,0001	0	0
genus	13	P	0	0,0001	0
genus	14	4	0	0	0,0001
genus	14	7	0	0,0001	0
genus	14	8	0	0	0
genus	1	1	0,0015	0	0
genus	1	2	0,0053	0	0
genus	1	3	0,0307	0	0,0003
genus	1	4	0,0163	0,0003	0,0004
genus	1	5	0,0033	0	0,0001
genus	1	6	0,0008	0	0
genus	1	7	0,0035	0	0
genus	1	8	0,0009	0	0
genus	1	9	0,0026	0	0
genus	1	10	0,0016	0	0
genus	1	P	0,0001	0	0
genus	2	1	0	0	0,0001
genus	2	2	0	0,0001	0
genus	2	5	0	0,0001	0
genus	2	7	0	0	0,0001
genus	2	9	0	0	0,0001
genus	2	10	0	0,0001	0
genus	2	P	0	0	0,0001
genus	6C1	3	0,0001	0	0
genus	6C1	6	0,0002	0	0
genus	7	1	0	0	0,0001
genus	7	2	0	0	0,0001
genus	7	3	0	0	0,0001
genus	7	7	0,0022	0	0
genus	7	P	0,0001	0	0
genus	9	2	0,0001	0	0
genus	Achromobacter	1	0,0026	0	0
genus	Achromobacter	2	0,0062	0	0
genus	Achromobacter	3	0,0014	0	0
genus	Achromobacter	4	0,0004	0	0
genus	Achromobacter	6	0,0022	0	0
genus	Achromobacter	7	0,0043	0	0
genus	Achromobacter	8	0,0007	0	0
genus	Achromobacter	9	0,0039	0	0
genus	Achromobacter	10	0,0038	0	0
genus	Acidibacter	1	0,0009	0	0

genus	Acinetobacter	1	0,0063	0,0076	0,0071
genus	Acinetobacter	2	0,0442	0,0059	0,0058
genus	Acinetobacter	3	0,0024	0,0081	0,0059
genus	Acinetobacter	4	0,0084	0,0065	0,0041
genus	Acinetobacter	5	0	0,0045	0,004
genus	Acinetobacter	6	0,0077	0,0051	0,0042
genus	Acinetobacter	7	0,0475	0,0193	0,0061
genus	Acinetobacter	8	0,0232	0,0046	0,0065
genus	Acinetobacter	9	0,0114	0,0045	0,0049
genus	Acinetobacter	10	0,0243	0,0037	0,0037
genus	Acinetobacter	P	0,0058	0,0078	0,0058
genus	Actinobacillus	2	0,0018	0	0
genus	Actinobacillus	3	0,0005	0	0
genus	Actinobacillus	4	0,0001	0	0
genus	Actinobacillus	6	0,0008	0	0,0001
genus	Actinobacillus	9	0	0,0001	0
genus	Actinobacillus	P	0	0	0
genus	Actinomyces	1	0,0006	0,0001	0
genus	Actinomyces	2	0,0004	0,0001	0,0001
genus	Actinomyces	4	0,0015	0,0001	0
genus	Actinomyces	5	0,0003	0,0001	0
genus	Actinomyces	6	0,0006	0	0
genus	Actinomyces	7	0	0	0,0001
genus	Actinomyces	8	0	0	0,0001
genus	Actinomyces	9	0	0	0,0001
genus	Actinomyces	10	0,0003	0	0
genus	Actinomyces	P	0,0033	0	0,0001
genus	Aeromonas	6	0,0001	0	0
genus	Aeromonas	8	0,0052	0	0
genus	Aeromonas	10	0,0002	0	0
genus	Aeromonas	P	0	0,0001	0
genus	Aggregatibacter	2	0,0011	0	0
genus	Aggregatibacter	6	0,0001	0	0
genus	Aggregatibacter	8	0	0,0001	0,0001
genus	Aggregatibacter	10	0,0035	0	0
genus	Aggregatibacter	P	0,0003	0	0
genus	Akkermansia	4	0,0006	0	0
genus	Alistipes	5	0,0001	0	0
genus	Alloprevotella	1	0	0	0,0002
genus	Alloprevotella	2	0,0008	0	0,0001
genus	Alloprevotella	4	0,0002	0,0001	0
genus	Alloprevotella	5	0	0,0003	0
genus	Alloprevotella	6	0	0	0,0001
genus	Alloprevotella	7	0,0041	0	0,0001
genus	Alloprevotella	8	0	0,0002	0,0001
genus	Alloprevotella	10	0	0,0001	0
genus	Alloprevotella	P	0,0001	0,0001	0
genus	Anaerococcus	2	0,0007	0	0
genus	Anaerococcus	3	0	0,0001	0
genus	Anaerococcus	5	0,0002	0	0
genus	Anaerococcus	6	0	0	0,0004
genus	Anaerococcus	7	0,003	0	0
genus	Anaerococcus	8	0	0	0
genus	Anaerococcus	9	0,0005	0	0
genus	Anaerococcus	10	0,0003	0	0

genus	Aquabacterium	2	0,0021	0	0
genus	Aquabacterium	6	0	0	0
genus	Aquabacterium	7	0	0,0001	0
genus	Aquabacterium	8	0,0008	0	0
genus	Aquabacterium	10	0,0004	0	0
genus	Aquabacterium	P	0,0001	0	0
genus	Arthrobacter	1	0,0002	0	0
genus	Arthrobacter	2	0,0001	0	0
genus	Arthrobacter	5	0,0002	0	0
genus	Arthrobacter	6	0,0003	0	0
genus	Arthrobacter	7	0,0002	0	0
genus	Arthrobacter	8	0,0001	0	0
genus	Arthrobacter	9	0,0005	0	0
genus	Arthrobacter	10	0,0002	0	0
genus	Atopobium	1	0,0015	0	0,0001
genus	Atopobium	2	0,001	0	0
genus	Atopobium	3	0,0003	0	0,0001
genus	Atopobium	4	0,0004	0,0001	0,0007
genus	Atopobium	5	0,001	0	0,0004
genus	Atopobium	6	0,0029	0	0
genus	Atopobium	7	0,0007	0	0
genus	Atopobium	8	0,0014	0	0,0001
genus	Atopobium	9	0,0008	0	0,0001
genus	Atopobium	10	0,0014	0	0,0001
genus	Aureimonas	9	0,0001	0	0
genus	Azospira	6	0,0001	0	0
genus	Bacillus	1	0,0006	0,0001	0
genus	Bacillus	2	0,0006	0,0001	0,0001
genus	Bacillus	3	0,0005	0,0001	0,0001
genus	Bacillus	4	0,0003	0,0002	0,0004
genus	Bacillus	5	0,0003	0,0002	0,0001
genus	Bacillus	6	0,0008	0	0,0001
genus	Bacillus	7	0,0001	0,0001	0
genus	Bacillus	8	0,0005	0,0001	0,0001
genus	Bacillus	9	0,0004	0	0,0001
genus	Bacillus	10	0,001	0,0001	0,0001
genus	Bacillus	P	0,0001	0,0001	0,0001
genus	bacterium	2	0,0012	0	0
genus	bacterium	3	0,0054	0	0,0001
genus	bacterium	4	0,0022	0	0
genus	bacterium	5	0,0004	0	0
genus	bacterium	6	0,0001	0	0
genus	bacterium	7	0,0001	0	0
genus	bacterium	8	0,0022	0	0
genus	bacterium	9	0,0006	0	0
genus	bacterium	10	0,0001	0	0
genus	Bartonella	3	0	0,0001	0
genus	Bartonella	7	0	0,0001	0
genus	Bartonella	9	0,0001	0	0
genus	Bergeyella	6	0,0005	0	0
genus	Bifidobacterium	5	0,0001	0	0
genus	Bifidobacterium	6	0,0018	0	0
genus	Bifidobacterium	9	0,0005	0	0
genus	Blautia	2	0,0001	0	0
genus	Blautia	5	0,0001	0	0

genus	Blautia	6	0,0001	0	0
genus	Blautia	7	0,0001	0	0
genus	Brachybacterium	7	0,0005	0	0
genus	Bradyrhizobium	2	0,0005	0	0
genus	Bradyrhizobium	3	0,001	0	0
genus	Bradyrhizobium	4	0,0003	0	0
genus	Bradyrhizobium	5	0,0001	0	0
genus	Brevibacterium	5	0,0003	0	0
genus	Brevibacterium	6	0,0015	0	0
genus	Brevibacterium	9	0,0017	0	0
genus	Brevundimonas	2	0,0011	0	0
genus	Brevundimonas	5	0	0,0001	0
genus	Brevundimonas	7	0	0	0,0001
genus	Brevundimonas	8	0,0013	0	0
genus	Brevundimonas	9	0,0015	0	0
genus	Brevundimonas	10	0,0001	0	0
genus	Brevundimonas	P	0	0,0001	0
genus	Campylobacter	7	0,0033	0	0
genus	Cardiobacterium	7	0,0019	0	0
genus	Cardiobacterium	P	0,0001	0	0
genus	Carnobacterium	2	0,0006	0	0
genus	Carnobacterium	5	0,0002	0	0
genus	Caulobacter	2	0,0014	0	0
genus	Chryseobacterium	1	0,0001	0	0
genus	Chryseobacterium	2	0,0032	0	0
genus	Chryseobacterium	4	0,0003	0	0
genus	Chryseobacterium	7	0,003	0	0
genus	Chryseobacterium	9	0,0002	0	0
genus	Chryseobacterium	10	0,0017	0	0
genus	Citrobacter	1	0	0,001	0,0009
genus	Citrobacter	2	0	0,0008	0,0009
genus	Citrobacter	3	0	0,001	0,0009
genus	Citrobacter	4	0	0,0009	0,0006
genus	Citrobacter	5	0	0,0011	0,0009
genus	Citrobacter	6	0,0001	0,0009	0,0011
genus	Citrobacter	7	0	0,0007	0,001
genus	Citrobacter	8	0	0,0008	0,001
genus	Citrobacter	9	0	0,001	0,001
genus	Citrobacter	10	0	0,0009	0,001
genus	Citrobacter	P	0,0008	0,0009	0,0011
genus	Cloacibacterium	2	0,0002	0	0
genus	Cloacibacterium	4	0,0001	0	0
genus	Cloacibacterium	7	0,0018	0	0
genus	Cloacibacterium	9	0,0007	0	0
genus	CM45	2	0	0	0,0001
genus	Corynebacterium	1	0,001	0	0
genus	Corynebacterium	2	0,0051	0	0
genus	Corynebacterium	3	0,1747	0,0004	0,003
genus	Corynebacterium	4	0,0896	0,0014	0,0024
genus	Corynebacterium	5	0,0098	0,0002	0,0006
genus	Corynebacterium	6	0,002	0,0004	0,0002
genus	Corynebacterium	7	0,0048	0,0001	0
genus	Corynebacterium	8	0,0079	0,0001	0,0001
genus	Corynebacterium	9	0,0086	0	0,0001
genus	Corynebacterium	10	0,001	0,0001	0

genus	Corynebacterium	P	0,0008	0	0
genus	Cronobacter	1	0,0002	0,0039	0,0024
genus	Cronobacter	2	0	0,0023	0,0026
genus	Cronobacter	3	0,0086	0,0042	0,1
genus	Cronobacter	4	0	0,0023	0,0016
genus	Cronobacter	5	0,0003	0,0026	0,006
genus	Cronobacter	6	0,0001	0,0028	0,0032
genus	Cronobacter	7	0	0,0029	0,0035
genus	Cronobacter	8	0	0,0033	0,0036
genus	Cronobacter	9	0	0,0049	0,0031
genus	Cronobacter	10	0	0,0026	0,003
genus	Cronobacter	P	0,0029	0,0032	0,0035
genus	Delftia	1	0	0,0003	0,0006
genus	Delftia	2	0	0,0002	0,0005
genus	Delftia	3	0	0,0007	0,0002
genus	Delftia	4	0	0,0003	0,0002
genus	Delftia	5	0,004	0,0002	0,0002
genus	Delftia	6	0	0,0005	0,0003
genus	Delftia	7	0	0,0006	0,0005
genus	Delftia	8	0	0,0004	0,0006
genus	Delftia	9	0	0,0002	0,0004
genus	Delftia	10	0	0,0005	0,0008
genus	Delftia	P	0,0032	0,0006	0,0005
genus	Devosia	1	0,0017	0	0
genus	Devosia	3	0,0004	0	0
genus	Devosia	6	0,001	0	0
genus	Diaminobutyricimona	2	0,0021	0	0
genus	Diaminobutyricimona	7	0,0012	0	0
genus	Dickeya	3	0,0001	0	0,0002
genus	Dickeya	5	0	0	0
genus	Dolosigranulum	5	0	0	0,0001
genus	Dolosigranulum	10	0,0006	0	0
genus	Dolosigranulum	P	0,0001	0	0
genus	Empedobacter	2	0,0006	0	0
genus	Empedobacter	9	0,0008	0	0
genus	Ensifer	6	0,0004	0	0
genus	Ensifer	9	0,0006	0	0
genus	Enterobacter	1	0,0128	0,4974	0,5194
genus	Enterobacter	2	0,0012	0,5146	0,5555
genus	Enterobacter	3	0,0107	0,5003	0,4825
genus	Enterobacter	4	0,0003	0,5258	0,3654
genus	Enterobacter	5	0,001	0,5107	0,6057
genus	Enterobacter	6	0,0112	0,5217	0,5105
genus	Enterobacter	7	0,0015	0,5023	0,6062
genus	Enterobacter	8	0,0004	0,5163	0,5188
genus	Enterobacter	9	0,0079	0,5233	0,5314
genus	Enterobacter	10	0,0004	0,5134	0,4919
genus	Enterobacter	P	0,57	0,525	0,5754
genus	Enterococcus	1	0	0,0055	0,0022
genus	Enterococcus	2	0	0,0038	0,0019
genus	Enterococcus	3	0	0,004	0,0021
genus	Enterococcus	4	0,059	0,0023	0,0061
genus	Enterococcus	5	0,0006	0,0022	0,003
genus	Enterococcus	6	0	0,0018	0,0021
genus	Enterococcus	7	0	0,0023	0,0031

genus	Enterococcus	8	0	0,0034	0,0034
genus	Enterococcus	9	0	0,0017	0,0022
genus	Enterococcus	10	0,0025	0,0019	0,0011
genus	Enterococcus	P	0,0096	0,0101	0,0108
genus	Erwinia	1	0	0,0002	0,0001
genus	Erwinia	2	0	0,0001	0,0001
genus	Erwinia	3	0	0,0002	0,0001
genus	Erwinia	4	0	0,0002	0,0001
genus	Erwinia	5	0	0,0001	0,0002
genus	Erwinia	6	0	0,0001	0,0001
genus	Erwinia	7	0	0,0001	0,0001
genus	Erwinia	8	0	0,0001	0,0002
genus	Erwinia	9	0	0,0002	0,0002
genus	Erwinia	10	0	0,0002	0,0001
genus	Erwinia	P	0,0001	0,0001	0,0002
genus	Exiguobacterium	1	0	0,0001	0
genus	Exiguobacterium	3	0	0	0
genus	Exiguobacterium	6	0	0	0
genus	Exiguobacterium	9	0	0	0
genus	Exiguobacterium	P	0	0	0,0001
genus	Falsirhodobacter	6	0	0	0,0001
genus	Falsirhodobacter	8	0,0007	0	0
genus	Fastidiosipila	1	0,0009	0	0
genus	Fastidiosipila	2	0,0012	0	0
genus	Fastidiosipila	3	0,0002	0	0
genus	Fastidiosipila	4	0,0005	0,0001	0,0004
genus	Fastidiosipila	5	0,0009	0	0,0002
genus	Fastidiosipila	6	0,0016	0	0
genus	Fastidiosipila	7	0,001	0	0
genus	Fastidiosipila	8	0,0016	0	0
genus	Fastidiosipila	9	0,0004	0	0
genus	Fastidiosipila	10	0,0018	0	0
genus	Fibrobacter	1	0,0001	0	0
genus	Finegoldia	1	0,0018	0	0
genus	Finegoldia	3	0,0008	0	0
genus	Finegoldia	9	0,0027	0	0
genus	Flavobacterium	4	0,0001	0	0
genus	Flavobacterium	6	0,0001	0	0
genus	Flavobacterium	7	0	0,0001	0
genus	Flavobacterium	9	0,0006	0	0
genus	Friedmanniella	2	0,0008	0	0
genus	Friedmanniella	3	0,0001	0	0
genus	Fusobacterium	3	0,0003	0,0001	0
genus	Fusobacterium	7	0,0025	0	0
genus	Fusobacterium	8	0,001	0	0
genus	Fusobacterium	10	0,0007	0	0
genus	Gemella	1	0,0009	0,0004	0,0002
genus	Gemella	2	0,0026	0,0003	0,0002
genus	Gemella	3	0,0006	0,0002	0,0003
genus	Gemella	4	0	0,0001	0,0001
genus	Gemella	5	0	0,0005	0,0001
genus	Gemella	6	0	0,0002	0,0001
genus	Gemella	7	0,0102	0,0001	0,0002
genus	Gemella	8	0,0003	0,0002	0,0002
genus	Gemella	9	0	0,0001	0,0003

genus	Gemella	10	0,0015	0,0002	0,0001
genus	Gemella	P	0,0018	0,0003	0,0002
genus	Gemmobacter	1	0,0007	0	0
genus	Gemmobacter	3	0,0008	0	0
genus	Gemmobacter	4	0,0009	0	0
genus	Gemmobacter	5	0,0003	0	0
genus	Gemmobacter	6	0,0001	0	0
genus	Gemmobacter	7	0,0048	0	0
genus	Gemmobacter	8	0,0081	0	0
genus	Gemmobacter	9	0,0015	0	0
genus	Gemmobacter	10	0,0008	0	0
genus	Geobacillus	8	0,0003	0	0
genus	Granulicatella	1	0,0006	0,0006	0,0005
genus	Granulicatella	2	0,0012	0,0002	0,0001
genus	Granulicatella	3	0,001	0,0006	0,0004
genus	Granulicatella	4	0	0,0002	0,0002
genus	Granulicatella	5	0	0,0004	0,0002
genus	Granulicatella	6	0,0006	0,0003	0,0001
genus	Granulicatella	7	0	0,0002	0,0003
genus	Granulicatella	8	0,0014	0,0002	0,0002
genus	Granulicatella	9	0,0021	0	0,0001
genus	Granulicatella	10	0,0027	0,0002	0,0002
genus	Granulicatella	P	0,0008	0,0003	0,0001
genus	group	1	0,0001	0	0
genus	group	2	0,0001	0	0
genus	group	5	0,0001	0	0
genus	group	7	0,0001	0	0
genus	group	8	0,0001	0	0
genus	Haemophilus	1	0,0041	0,0001	0,0002
genus	Haemophilus	2	0,0079	0,0002	0,0001
genus	Haemophilus	3	0,0009	0,0002	0,0002
genus	Haemophilus	4	0,0011	0	0,0002
genus	Haemophilus	5	0,0004	0,0004	0,0003
genus	Haemophilus	6	0,0039	0,0001	0,0002
genus	Haemophilus	7	0,0087	0,0001	0,0002
genus	Haemophilus	8	0,0033	0,0003	0,0011
genus	Haemophilus	9	0,0062	0,0001	0,0002
genus	Haemophilus	10	0,0121	0	0,0002
genus	Haemophilus	P	0,0226	0,0002	0,0001
genus	Hydrocarboniphaga	1	0	0,0001	0
genus	Hydrocarboniphaga	2	0	0,0001	0,0001
genus	Hydrocarboniphaga	3	0	0,0001	0,0001
genus	Hydrocarboniphaga	6	0	0	0,0001
genus	Hydrocarboniphaga	7	0	0,0001	0
genus	Hydrocarboniphaga	9	0	0	0
genus	Hydrocarboniphaga	10	0	0	0,0001
genus	Hydrocarboniphaga	P	0	0	0,0001
genus	Intestinibacter	1	0,0006	0	0
genus	Intestinibacter	2	0,0001	0	0
genus	Intestinibacter	4	0,0002	0	0
genus	Intestinibacter	9	0,0001	0	0
genus	Jeotgalicoccus	2	0,0013	0	0
genus	Jeotgalicoccus	10	0,0005	0	0
genus	Klebsiella	1	0,0004	0,032	0,0209
genus	Klebsiella	2	0,0001	0,0195	0,0192

genus	Klebsiella	3	0,0003	0,0238	0,0199
genus	Klebsiella	4	0,0003	0,0189	0,0122
genus	Klebsiella	5	0	0,0185	0,0243
genus	Klebsiella	6	0,0007	0,0211	0,0186
genus	Klebsiella	7	0	0,0184	0,0218
genus	Klebsiella	8	0	0,0177	0,0198
genus	Klebsiella	9	0,0004	0,0263	0,0191
genus	Klebsiella	10	0	0,0181	0,0173
genus	Klebsiella	P	0,0171	0,0206	0,0216
genus	Knoellia	2	0,0006	0	0
genus	Knoellia	9	0,0001	0	0
genus	Kocuria	1	0,002	0	0
genus	Kocuria	2	0,0026	0,0001	0
genus	Kocuria	3	0,0013	0	0
genus	Kocuria	4	0,0004	0	0
genus	Kocuria	6	0,0025	0	0
genus	Kocuria	7	0,0039	0	0
genus	Kocuria	8	0	0	0
genus	Kocuria	9	0,0026	0	0
genus	Kocuria	10	0,0011	0	0
genus	Kocuria	P	0	0	0
genus	Lactobacillus	1	0,0022	0,0002	0
genus	Lactobacillus	2	0,002	0,0001	0
genus	Lactobacillus	3	0,0003	0	0
genus	Lactobacillus	4	0,0009	0	0
genus	Lactobacillus	5	0,0013	0,0001	0,0001
genus	Lactobacillus	6	0,0075	0	0
genus	Lactobacillus	8	0,0078	0,0001	0
genus	Lactobacillus	9	0,0038	0,0001	0
genus	Lactobacillus	10	0,0032	0	0,0001
genus	Lactococcus	1	0,001	0	0
genus	Lactococcus	2	0,0034	0	0
genus	Lactococcus	5	0,0002	0	0
genus	Lactococcus	6	0,0001	0	0
genus	Lactococcus	9	0,0031	0	0
genus	Lactococcus	10	0,0015	0,0001	0
genus	Lactococcus	P	0,0006	0	0
genus	Leptotrichia	5	0	0,0001	0
genus	Leptotrichia	7	0,0014	0	0
genus	Leptotrichia	10	0	0	0,0001
genus	Leucobacter	1	0,0005	0	0
genus	Leucobacter	2	0,0006	0	0
genus	Leucobacter	3	0,0001	0	0
genus	Leucobacter	6	0,0001	0	0
genus	Leucobacter	7	0,0006	0	0,0001
genus	Leucobacter	9	0,0001	0	0
genus	Leuconostoc	3	0,0005	0	0
genus	Lysinibacillus	1	0,0004	0	0
genus	Lysinibacillus	2	0,0013	0	0
genus	Lysinibacillus	3	0,0005	0	0
genus	Lysinibacillus	4	0,0003	0	0
genus	Lysinibacillus	5	0	0,0001	0
genus	Lysinibacillus	6	0	0,0001	0,0001
genus	Lysinibacillus	9	0	0	0
genus	Lysinibacillus	P	0	0,0001	0

genus	Massilia	4	0,0003	0	0
genus	Massilia	5	0,0001	0	0
genus	Massilia	7	0	0	0,0001
genus	Massilia	9	0,0007	0	0
genus	Megasphaera	P	0,0004	0	0
genus	Mesorhizobium	4	0	0,0001	0
genus	Mesorhizobium	5	0,0004	0	0
genus	Mesorhizobium	6	0,0002	0	0
genus	Mesorhizobium	7	0	0,0001	0
genus	Mesorhizobium	9	0	0	0,0001
genus	Mesorhizobium	10	0	0	0,0001
genus	Mesorhizobium	P	0,0001	0,0001	0
genus	Methanobrevibacter	4	0,0004	0	0
genus	Methylobacterium	2	0,003	0	0
genus	Methylobacterium	4	0,0002	0	0
genus	Methylobacterium	6	0,0003	0	0
genus	Methylobacterium	7	0,0013	0	0
genus	Methylobacterium	9	0,0002	0	0
genus	Methylobacterium	10	0,0005	0	0
genus	Methylocella	1	0,0003	0	0
genus	Methylocella	7	0,0015	0	0
genus	Microbacterium	1	0,0003	0,0001	0,0001
genus	Microbacterium	2	0,0007	0,0002	0,0001
genus	Microbacterium	3	0	0,0001	0
genus	Microbacterium	4	0	0	0,0001
genus	Microbacterium	6	0	0,0001	0
genus	Microbacterium	7	0,003	0	0
genus	Microbacterium	8	0,0007	0,0001	0,0001
genus	Microbacterium	10	0,0014	0	0
genus	Microbacterium	P	0,0001	0	0,0001
genus	Micrococcus	1	0,0001	0	0
genus	Micrococcus	2	0	0,0001	0
genus	Micrococcus	3	0,0013	0	0
genus	Micrococcus	4	0,0008	0	0
genus	Micrococcus	7	0,0018	0	0
genus	Micrococcus	9	0,0008	0	0
genus	Micrococcus	10	0,001	0	0
genus	Microvirga	2	0,0108	0	0
genus	Mogibacterium	1	0,0003	0	0
genus	Mogibacterium	4	0,0001	0	0
genus	Moraxella	1	0,0294	0,0001	0,0002
genus	Moraxella	2	0,1001	0,0001	0,0001
genus	Moraxella	3	0,0127	0,0002	0
genus	Moraxella	4	0,0184	0,0001	0
genus	Moraxella	5	0,0047	0,0001	0,0002
genus	Moraxella	6	0,0538	0,0001	0,0006
genus	Moraxella	7	0,2201	0	0,0003
genus	Moraxella	8	0,0441	0,0002	0,0006
genus	Moraxella	9	0,0495	0,0001	0,0005
genus	Moraxella	10	0,058	0,0003	0,0001
genus	Moraxella	P	0,0013	0,0018	0,0001
genus	Neisseria	1	0	0,0005	0,0001
genus	Neisseria	2	0,0036	0,0001	0,0001
genus	Neisseria	3	0	0,0003	0,0001
genus	Neisseria	4	0,0001	0,0001	0,0018

genus	Neisseria	5	0	0,0002	0
genus	Neisseria	6	0	0,0001	0,0001
genus	Neisseria	7	0,002	0	0
genus	Neisseria	8	0	0,0007	0,0014
genus	Neisseria	9	0	0	0,0001
genus	Neisseria	10	0	0,0001	0,0001
genus	Neisseria	P	0,0007	0,0003	0,0001
genus	Nesterenkonia	6	0,0001	0	0
genus	Nevskia	2	0,0004	0	0
genus	Nevskia	3	0,0003	0	0
genus	Nevskia	4	0,0001	0	0
genus	Nevskia	5	0,0003	0	0
genus	Nevskia	6	0,001	0	0
genus	Nevskia	7	0,0017	0	0
genus	Nevskia	8	0,0004	0	0
genus	Novosphingobium	4	0,0002	0	0
genus	Novosphingobium	7	0	0,0001	0,0001
genus	Other	1	0,0003	0	0
genus	Other	2	0,0004	0	0
genus	Other	3	0,0001	0	0
genus	Other	4	0,0001	0	0
genus	Other	6	0,0002	0	0
genus	Other	7	0,0002	0	0
genus	Other	8	0,0002	0	0
genus	Other	9	0,0004	0	0
genus	Other	10	0,0001	0	0
genus	Paenibacillus	1	0	0,0005	0,0002
genus	Paenibacillus	2	0	0,0003	0,0002
genus	Paenibacillus	3	0	0,0002	0,0001
genus	Paenibacillus	4	0	0,0001	0,0002
genus	Paenibacillus	5	0	0,0004	0,0002
genus	Paenibacillus	6	0,0001	0,0001	0,0003
genus	Paenibacillus	7	0	0,0001	0,0002
genus	Paenibacillus	8	0	0,0001	0,0002
genus	Paenibacillus	9	0	0,0003	0,0001
genus	Paenibacillus	10	0,0001	0,0001	0,0002
genus	Paenibacillus	P	0,0003	0,0003	0,0002
genus	Paenisporosarcina	1	0,0004	0	0
genus	Paenisporosarcina	2	0,0012	0	0
genus	Paenisporosarcina	3	0,0004	0	0
genus	Paenisporosarcina	4	0,0002	0	0
genus	Pantoea	1	0,0021	0,0456	0,05
genus	Pantoea	2	0	0,0523	0,0473
genus	Pantoea	3	0,0003	0,0557	0,0416
genus	Pantoea	4	0,0003	0,0433	0,0273
genus	Pantoea	5	0,0001	0,0602	0,021
genus	Pantoea	6	0,0006	0,0604	0,0664
genus	Pantoea	7	0	0,0654	0,0393
genus	Pantoea	8	0,0001	0,0774	0,0612
genus	Pantoea	9	0,0004	0,067	0,0594
genus	Pantoea	10	0	0,0622	0,0632
genus	Pantoea	P	0,0477	0,0599	0,0506
genus	Paracocccus	1	0,0007	0	0
genus	Paracocccus	2	0,001	0	0
genus	Paracocccus	3	0,0002	0,0001	0

genus	Paracocccus	4	0,0003	0	0
genus	Paracocccus	5	0,0008	0	0
genus	Paracocccus	6	0,0035	0	0,0001
genus	Paracocccus	7	0,0017	0	0
genus	Paracocccus	8	0,0036	0	0
genus	Paracocccus	9	0,0008	0	0,0001
genus	Paracocccus	10	0,0003	0	0
genus	Pectobacterium	1	0,0001	0,0077	0,0061
genus	Pectobacterium	2	0	0,0062	0,0051
genus	Pectobacterium	3	0,0002	0,0067	0,0103
genus	Pectobacterium	4	0	0,0063	0,0035
genus	Pectobacterium	5	0,0001	0,0062	0,0027
genus	Pectobacterium	6	0,0004	0,0059	0,0056
genus	Pectobacterium	7	0	0,0052	0,0044
genus	Pectobacterium	8	0	0,0055	0,0053
genus	Pectobacterium	9	0,0001	0,0053	0,005
genus	Pectobacterium	10	0	0,0053	0,0045
genus	Pectobacterium	P	0,0041	0,006	0,0058
genus	Pediococcus	6	0,0011	0	0
genus	Pediococcus	7	0,0009	0	0
genus	Pediococcus	8	0	0,0001	0
genus	Pediococcus	9	0	0	0
genus	Peptoclostridium	2	0,002	0	0
genus	Peptoclostridium	4	0,001	0	0
genus	Peptoclostridium	9	0,0026	0	0
genus	Peptoniphilus	3	0,0007	0	0
genus	Peptoniphilus	5	0	0,0001	0
genus	Peptoniphilus	10	0,0004	0	0
genus	Petrimonas	1	0,0003	0	0
genus	Petrimonas	6	0	0	0,0001
genus	Phenylobacterium	2	0,0002	0	0
genus	Phycicoccus	2	0,0003	0	0
genus	Phycicoccus	3	0,0001	0	0
genus	Phycicoccus	7	0,0001	0	0
genus	Phycicoccus	9	0,0001	0	0
genus	Phycicola	1	0,0001	0	0
genus	Phycicola	2	0,0001	0	0
genus	Phycicola	7	0,0001	0	0
genus	Planomicrobium	1	0,0001	0	0
genus	Planomicrobium	4	0,0001	0	0,0001
genus	Planomicrobium	5	0	0	0,0001
genus	Planomicrobium	6	0,0001	0	0
genus	Planomicrobium	7	0,0003	0	0
genus	Planomicrobium	9	0,0001	0	0
genus	Planomicrobium	10	0,0003	0	0
genus	Porphyromonas	P	0,0002	0	0
genus	Prevotella	5	0,0002	0,0001	0
genus	Propionibacterium	1	0,0578	0,0002	0,0003
genus	Propionibacterium	2	0,0864	0,0004	0,0006
genus	Propionibacterium	3	0,03	0,0006	0,0002
genus	Propionibacterium	4	0,0225	0,0002	0,0002
genus	Propionibacterium	5	0,015	0,0008	0,0017
genus	Propionibacterium	6	0,1199	0,0033	0,0047
genus	Propionibacterium	7	0,1496	0,0017	0,0009
genus	Propionibacterium	8	0,0287	0,0011	0,002

genus	Propionibacterium	9	0,144	0,0012	0,0019
genus	Propionibacterium	10	0,064	0,0019	0,0008
genus	Propionibacterium	P	0,0101	0,0021	0,0005
genus	Propioniciclava	4	0,0004	0	0
genus	proteobacterium	6	0,0013	0	0
genus	proteobacterium	7	0,0001	0	0
genus	proteobacterium	8	0,0039	0	0
genus	proteobacterium	9	0,0002	0	0
genus	proteobacterium	10	0,0001	0	0
genus	proteobacterium	P	0	0,0001	0
genus	Proteus	1	0	0,0047	0,0067
genus	Proteus	2	0	0,0063	0,0074
genus	Proteus	3	0,0001	0,0049	0,006
genus	Proteus	4	0	0,0072	0,0044
genus	Proteus	5	0	0,0053	0,0099
genus	Proteus	6	0,0001	0,0046	0,0052
genus	Proteus	7	0	0,0048	0,0083
genus	Proteus	8	0	0,0037	0,0047
genus	Proteus	9	0	0,0044	0,0053
genus	Proteus	10	0	0,0045	0,0054
genus	Proteus	P	0,0064	0,0047	0,0068
genus	Pseudoalteromonas	2	0,0002	0	0
genus	Pseudoalteromonas	8	0,0002	0	0
genus	Pseudomonas	1	0,0135	0,1172	0,1023
genus	Pseudomonas	2	0,0519	0,1099	0,0874
genus	Pseudomonas	3	0,0075	0,1004	0,0748
genus	Pseudomonas	4	0,0066	0,0964	0,0636
genus	Pseudomonas	5	0,0007	0,0841	0,0679
genus	Pseudomonas	6	0,0401	0,077	0,0691
genus	Pseudomonas	7	0,0531	0,0841	0,1007
genus	Pseudomonas	8	0,0137	0,0755	0,0676
genus	Pseudomonas	9	0,0297	0,0779	0,0883
genus	Pseudomonas	10	0,0377	0,0679	0,0798
genus	Pseudomonas	P	0,0566	0,0841	0,0789
genus	Psychrobacter	2	0,0001	0	0
genus	Psychrobacter	10	0,0015	0	0
genus	Ralstonia	4	0,0002	0	0
genus	Ralstonia	7	0	0	0,0001
genus	Ralstonia	9	0,0006	0	0
genus	Raoultella	1	0	0,0002	0,0001
genus	Raoultella	2	0	0,0001	0,0001
genus	Raoultella	3	0	0,0002	0,0002
genus	Raoultella	4	0	0,0001	0
genus	Raoultella	5	0	0,0001	0,0001
genus	Raoultella	6	0	0,0001	0,0001
genus	Raoultella	7	0	0,0001	0,0001
genus	Raoultella	8	0	0,0001	0,0001
genus	Raoultella	9	0	0,0001	0,0001
genus	Raoultella	10	0	0,0001	0,0001
genus	Raoultella	P	0,0001	0,0001	0,0001
genus	Rathayibacter	6	0,0001	0	0
genus	Rathayibacter	10	0,0007	0	0
genus	Rhizobium	1	0	0,0006	0,0004
genus	Rhizobium	2	0,002	0,0004	0,0004
genus	Rhizobium	3	0,0025	0,0003	0,0005

genus	Rhizobium	4	0,0001	0,0005	0,0002
genus	Rhizobium	5	0,0005	0,0005	0,0002
genus	Rhizobium	6	0,0026	0,0006	0,0004
genus	Rhizobium	7	0,0006	0,001	0,0004
genus	Rhizobium	8	0,0037	0,0004	0,0004
genus	Rhizobium	9	0,0018	0,0004	0,0005
genus	Rhizobium	10	0	0,0002	0,0004
genus	Rhizobium	P	0,0007	0,0011	0,0005
genus	Rhodococcus	3	0	0	0,0002
genus	Rhodococcus	5	0	0	0
genus	Rhodococcus	10	0	0	0,0001
genus	Rhodococcus	P	0,0001	0	0
genus	Roseomonas	4	0,0005	0	0
genus	Rothia	1	0,0012	0,0008	0,0006
genus	Rothia	2	0,0017	0,0005	0,0004
genus	Rothia	3	0,0007	0,0007	0,0006
genus	Rothia	4	0,0002	0,0006	0,0004
genus	Rothia	5	0,032	0,0008	0,0013
genus	Rothia	6	0,0018	0,0005	0,0003
genus	Rothia	7	0,0028	0,0006	0,0005
genus	Rothia	8	0,0017	0,0003	0,0003
genus	Rothia	9	0,0038	0,0003	0,0003
genus	Rothia	10	0	0,0004	0,0004
genus	Rothia	P	0,0041	0,0004	0,0006
genus	Saccharopolyspora	4	0,0002	0	0
genus	Saccharopolyspora	10	0,0002	0	0
genus	Salmonella	1	0	0,0005	0,0003
genus	Salmonella	2	0	0,0003	0,0003
genus	Salmonella	3	0	0,0003	0,0003
genus	Salmonella	4	0	0,0003	0,0002
genus	Salmonella	5	0	0,0003	0,0005
genus	Salmonella	6	0	0,0004	0,0006
genus	Salmonella	7	0	0,0003	0,0004
genus	Salmonella	8	0	0,0004	0,0008
genus	Salmonella	9	0	0,0005	0,0004
genus	Salmonella	10	0	0,0003	0,0003
genus	Salmonella	P	0,0005	0,0004	0,0004
genus	Sediminibacterium	9	0,0032	0	0
genus	Sedis	1	0,001	0	0
genus	Sedis	2	0,0002	0	0
genus	Sedis	3	0,0001	0	0
genus	Sedis	4	0,0002	0	0,0002
genus	Sedis	5	0,0003	0	0,0001
genus	Sedis	6	0,0008	0	0
genus	Sedis	7	0,0002	0	0
genus	Sedis	8	0,0005	0	0,0001
genus	Sedis	9	0,0002	0	0
genus	Sedis	10	0,0004	0	0,0001
genus	Serratia	3	0,0004	0	0
genus	Serratia	7	0,0057	0	0
genus	Serratia	9	0,0006	0	0
genus	Shewanella	1	0	0,0006	0,0005
genus	Shewanella	2	0,0017	0,0008	0,0005
genus	Shewanella	3	0	0,0005	0,0002
genus	Shewanella	4	0	0,0007	0,0004

genus	Shewanella	5	0	0,0005	0,0001
genus	Shewanella	6	0	0,0005	0,0002
genus	Shewanella	7	0	0,0004	0,0004
genus	Shewanella	8	0,0016	0,0003	0,0003
genus	Shewanella	9	0	0,0002	0,0002
genus	Shewanella	10	0	0,0003	0,0004
genus	Shewanella	P	0,0002	0,0005	0,0004
genus	Shigella	1	0,0071	0,2494	0,2413
genus	Shigella	2	0,0002	0,2532	0,2224
genus	Shigella	3	0,0016	0,2673	0,1827
genus	Shigella	4	0,0003	0,2167	0,1342
genus	Shigella	5	0,0003	0,2748	0,0974
genus	Shigella	6	0,0084	0,2785	0,2464
genus	Shigella	7	0,0003	0,2723	0,1811
genus	Shigella	8	0,0002	0,2685	0,2453
genus	Shigella	9	0,0047	0,2711	0,2432
genus	Shigella	10	0,0002	0,2848	0,2519
genus	Shigella	P	0,1964	0,2483	0,217
genus	Shinella	10	0,0001	0	0
genus	Simplicispira	P	0,0001	0	0
genus	Solobacterium	8	0	0,0001	0
genus	Sphingobium	1	0,0005	0	0
genus	Sphingobium	2	0,0004	0	0
genus	Sphingobium	3	0,0002	0,0001	0
genus	Sphingobium	8	0	0,0001	0,0001
genus	Sphingobium	10	0	0,0001	0
genus	Sphingobium	P	0,0001	0,0001	0,0001
genus	Sphingomonas	1	0,0005	0	0
genus	Sphingomonas	2	0,001	0	0
genus	Sphingomonas	4	0,0006	0	0
genus	Sphingomonas	6	0	0	0,0001
genus	Sphingomonas	7	0,0016	0	0
genus	Sphingomonas	9	0,0001	0	0
genus	Sphingomonas	10	0,0012	0	0
genus	Sphingomonas	P	0	0,0001	0
genus	Sporosarcina	2	0,0001	0	0
genus	Sporosarcina	3	0,0001	0	0
genus	Sporosarcina	4	0,0001	0	0,0001
genus	Sporosarcina	5	0,0001	0	0
genus	Sporosarcina	6	0,0003	0	0
genus	Sporosarcina	8	0,0001	0	0
genus	Sporosarcina	9	0,0001	0	0
genus	Sporosarcina	10	0,0002	0	0
genus	Staphylococcus	1	0,7773	0,0069	0,0234
genus	Staphylococcus	2	0,5296	0,0104	0,032
genus	Staphylococcus	3	0,2195	0,0069	0,0549
genus	Staphylococcus	4	0,1941	0,0545	0,3537
genus	Staphylococcus	5	0,4672	0,0096	0,1387
genus	Staphylococcus	6	0,6577	0,0041	0,0511
genus	Staphylococcus	7	0,318	0,0082	0,004
genus	Staphylococcus	8	0,7499	0,0112	0,0431
genus	Staphylococcus	9	0,6032	0,003	0,0241
genus	Staphylococcus	10	0,7008	0,0228	0,0667
genus	Staphylococcus	P	0,0068	0,0096	0,0083
genus	Stenotrophomonas	1	0	0	0

genus	Stenotrophomonas	2	0,0001	0,0001	0
genus	Stenotrophomonas	4	0	0	0,0001
genus	Stenotrophomonas	5	0	0,0001	0
genus	Stenotrophomonas	6	0,0003	0,0001	0,0001
genus	Stenotrophomonas	7	0	0,0001	0
genus	Stenotrophomonas	9	0	0	0,0001
genus	Stenotrophomonas	P	0,0001	0,0001	0,0001
genus	Streptococcus	1	0,04	0,0112	0,0098
genus	Streptococcus	2	0,0351	0,0072	0,0063
genus	Streptococcus	3	0,0241	0,0064	0,006
genus	Streptococcus	4	0,0156	0,0074	0,0085
genus	Streptococcus	5	0,3948	0,0106	0,0086
genus	Streptococcus	6	0,0365	0,0063	0,005
genus	Streptococcus	7	0,048	0,006	0,0112
genus	Streptococcus	8	0,0363	0,0046	0,0081
genus	Streptococcus	9	0,0368	0,004	0,005
genus	Streptococcus	10	0,0401	0,0044	0,0042
genus	Streptococcus	P	0,0135	0,0076	0,0069
genus	Streptomyces	1	0,0001	0	0
genus	Streptomyces	2	0,0001	0	0
genus	Streptomyces	3	0,0002	0	0
genus	Streptomyces	6	0,0004	0	0
genus	Streptomyces	7	0,0002	0	0
genus	Streptomyces	9	0,0003	0	0
genus	Streptomyces	10	0,0001	0	0
genus	Succiniclasticum	5	0,0002	0	0
genus	Succiniclasticum	10	0	0	0,0001
genus	Tatumella	1	0	0,0001	0,0001
genus	Tatumella	2	0	0,0001	0
genus	Tatumella	3	0	0,0001	0,0001
genus	Tatumella	4	0	0,0001	0
genus	Tatumella	5	0	0,0001	0,0001
genus	Tatumella	6	0	0,0001	0,0001
genus	Tatumella	7	0	0,0001	0,0001
genus	Tatumella	8	0	0,0001	0,0001
genus	Tatumella	9	0	0,0002	0,0001
genus	Tatumella	10	0	0,0001	0,0001
genus	Tatumella	P	0,0001	0,0001	0,0001
genus	Tepidimonas	2	0,0009	0	0
genus	Tepidimonas	3	0,0005	0	0
genus	Tepidimonas	6	0,0002	0	0
genus	Tepidimonas	7	0,0025	0	0
genus	Terrabacter	2	0,001	0	0
genus	Terrabacter	9	0,0001	0	0
genus	Thauera	4	0,0003	0	0
genus	Thauera	6	0	0	0,0001
genus	Trabulsiella	1	0	0,0001	0
genus	Trabulsiella	2	0	0,0001	0
genus	Trabulsiella	5	0	0	0,0001
genus	Trabulsiella	6	0	0	0
genus	Trabulsiella	9	0	0,0001	0
genus	Trabulsiella	10	0	0,0001	0
genus	Trabulsiella	P	0,0001	0	0
genus	Trichococcus	3	0,0001	0	0
genus	Trichococcus	5	0,0001	0	0

genus	tropica	2	0	0	0,0001
genus	Turicibacter	1	0,0031	0	0
genus	Turicibacter	2	0	0	0,0001
genus	uncultured	1	0,0004	0	0
genus	uncultured	2	0,0014	0	0
genus	uncultured	3	0,0002	0	0
genus	uncultured	4	0,0002	0	0
genus	uncultured	5	0,0001	0	0
genus	uncultured	6	0,0005	0	0
genus	uncultured	7	0,0019	0	0
genus	uncultured	8	0,0003	0	0
genus	uncultured	9	0,0013	0	0
genus	uncultured	10	0,0009	0	0
genus	uncultured	P	0,0001	0	0
genus	Veillonella	1	0,0016	0,002	0,0012
genus	Veillonella	2	0,0051	0,0012	0,0008
genus	Veillonella	3	0,2656	0,0023	0,0014
genus	Veillonella	4	0,4504	0,003	0,0027
genus	Veillonella	5	0,0354	0,0015	0,0008
genus	Veillonella	6	0,0015	0,001	0,0004
genus	Veillonella	7	0,0181	0,0005	0,0027
genus	Veillonella	8	0	0,0004	0,0006
genus	Veillonella	9	0,009	0,0005	0,0006
genus	Veillonella	10	0,0034	0,0008	0,0005
genus	Veillonella	P	0,0066	0,0011	0,0015
genus	Vibrio	1	0	0,0004	0,0002
genus	Vibrio	2	0	0,0003	0,0001
genus	Vibrio	3	0	0,0003	0,0002
genus	Vibrio	4	0	0,0002	0,0002
genus	Vibrio	5	0	0,0001	0
genus	Vibrio	6	0	0,0001	0,0002
genus	Vibrio	7	0	0,0001	0,0001
genus	Vibrio	8	0	0,0001	0,0001
genus	Vibrio	9	0	0	0,0002
genus	Vibrio	10	0	0,0001	0,0001
genus	Vibrio	P	0,0001	0,0002	0,0002
genus	Weeksella	9	0,0003	0	0
genus	Weissella	1	0,0023	0	0
genus	Weissella	3	0,0005	0	0
genus	Weissella	6	0,002	0	0
genus	Wolbachia	3	0	0,0001	0
genus	XI	1	0,0013	0,0002	0,0001
genus	XI	2	0,0017	0,0001	0,0001
genus	XI	3	0,0011	0,0002	0,0001
genus	XI	4	0	0	0,0001
genus	XI	5	0,0001	0,0003	0
genus	XI	6	0	0,0001	0,0002
genus	XI	7	0,0066	0	0,0001
genus	XI	8	0,0001	0,0001	0,0001
genus	XI	9	0,0016	0,0001	0,0002
genus	XI	10	0,0011	0,0001	0
genus	XI	P	0,0009	0,0001	0,0001
genus	XII	1	0,001	0,0001	0
genus	XII	2	0,0004	0	0
genus	XII	3	0,0001	0	0,0001

genus	XII	4	0,0003	0,0001	0,0004
genus	XII	5	0,0005	0	0,0002
genus	XII	6	0,0016	0	0
genus	XII	7	0,0003	0	0
genus	XII	8	0,001	0	0,0002
genus	XII	9	0,0005	0	0
genus	XII	10	0,0008	0	0,0001
genus	XII	P	0	0	0,0001
genus	XIII	1	0,0003	0	0
genus	XIII	4	0,0001	0	0
family	Acetobacteraceae	4	0,0005	0	0
family	Acidaminococcaceae	5	0,0002	0	0
family	Acidaminococcaceae	10	0	0	0,0001
family	Actinomycetaceae	1	0,0006	0,0001	0
family	Actinomycetaceae	2	0,0004	0,0001	0,0001
family	Actinomycetaceae	4	0,0015	0,0001	0
family	Actinomycetaceae	5	0,0003	0,0001	0
family	Actinomycetaceae	6	0,0006	0	0
family	Actinomycetaceae	7	0	0	0,0001
family	Actinomycetaceae	8	0	0	0,0001
family	Actinomycetaceae	9	0	0	0,0001
family	Actinomycetaceae	10	0,0003	0	0
family	Actinomycetaceae	P	0,0033	0	0,0001
family	Aeromonadaceae	6	0,0001	0	0
family	Aeromonadaceae	8	0,0052	0	0
family	Aeromonadaceae	10	0,0002	0	0
family	Aeromonadaceae	P	0	0,0001	0
family	Alcaligenaceae	1	0,0051	0	0
family	Alcaligenaceae	2	0,012	0	0
family	Alcaligenaceae	3	0,0021	0	0
family	Alcaligenaceae	4	0,0009	0	0
family	Alcaligenaceae	6	0,0027	0	0
family	Alcaligenaceae	7	0,0098	0	0
family	Alcaligenaceae	8	0,0016	0	0
family	Alcaligenaceae	9	0,0078	0	0
family	Alcaligenaceae	10	0,008	0	0
family	Anaplasmataceae	3	0	0,0001	0
family	Aurantimonadaceae	9	0,0001	0	0
family	Bacillaceae	1	0,0006	0,0001	0
family	Bacillaceae	2	0,0006	0,0001	0,0001
family	Bacillaceae	3	0,0005	0,0001	0,0001
family	Bacillaceae	4	0,0003	0,0002	0,0004
family	Bacillaceae	5	0,0003	0,0002	0,0001
family	Bacillaceae	6	0,0008	0	0,0001
family	Bacillaceae	7	0,0001	0,0001	0
family	Bacillaceae	8	0,0009	0,0001	0,0001
family	Bacillaceae	9	0,0004	0	0,0001
family	Bacillaceae	10	0,001	0,0001	0,0001
family	Bacillaceae	P	0,0001	0,0001	0,0001
family	bacterium	1	0,001	0	0
family	bacterium	2	0,0026	0	0
family	bacterium	3	0,0005	0	0
family	bacterium	8	0,0001	0	0
family	bacterium	9	0,0001	0	0
family	Bartonellaceae	3	0	0,0001	0

family	Bartonellaceae	7	0	0,0001	0
family	Bartonellaceae	9	0,0001	0	0
family	Beijerinckiaceae	1	0,0003	0	0
family	Beijerinckiaceae	7	0,0015	0	0
family	Bifidobacteriaceae	5	0,0001	0	0
family	Bifidobacteriaceae	6	0,0018	0	0
family	Bifidobacteriaceae	9	0,0005	0	0
family	Bradyrhizobiaceae	2	0,0005	0	0
family	Bradyrhizobiaceae	3	0,001	0	0
family	Bradyrhizobiaceae	4	0,0003	0	0
family	Bradyrhizobiaceae	5	0,0001	0	0
family	Brevibacteriaceae	5	0,0003	0	0
family	Brevibacteriaceae	6	0,0015	0	0
family	Brevibacteriaceae	9	0,0017	0	0
family	Burkholderiaceae	4	0,0002	0	0
family	Burkholderiaceae	7	0	0	0,0001
family	Burkholderiaceae	9	0,0006	0	0
family	Campylobacteraceae	7	0,0033	0	0
family	Cardiobacteriaceae	7	0,0019	0	0
family	Cardiobacteriaceae	P	0,0001	0	0
family	Carnobacteriaceae	1	0,0006	0,0006	0,0005
family	Carnobacteriaceae	2	0,0019	0,0002	0,0001
family	Carnobacteriaceae	3	0,0011	0,0006	0,0004
family	Carnobacteriaceae	4	0	0,0002	0,0002
family	Carnobacteriaceae	5	0,0003	0,0004	0,0002
family	Carnobacteriaceae	6	0,0006	0,0003	0,0001
family	Carnobacteriaceae	7	0	0,0002	0,0003
family	Carnobacteriaceae	8	0,0014	0,0002	0,0003
family	Carnobacteriaceae	9	0,0021	0,0001	0,0001
family	Carnobacteriaceae	10	0,0034	0,0002	0,0002
family	Carnobacteriaceae	P	0,0009	0,0003	0,0002
family	Caulobacteraceae	2	0,0029	0	0
family	Caulobacteraceae	5	0	0,0001	0
family	Caulobacteraceae	7	0	0	0,0001
family	Caulobacteraceae	8	0,0019	0	0
family	Caulobacteraceae	9	0,0015	0	0
family	Caulobacteraceae	10	0,0018	0	0
family	Caulobacteraceae	P	0	0,0001	0
family	Chitinophagaceae	9	0,0032	0	0
family	Christensenellaceae	1	0,0003	0	0
family	Comamonadaceae	1	0	0,0003	0,0006
family	Comamonadaceae	2	0,003	0,0002	0,0006
family	Comamonadaceae	3	0,0005	0,0007	0,0002
family	Comamonadaceae	4	0	0,0003	0,0002
family	Comamonadaceae	5	0,004	0,0002	0,0003
family	Comamonadaceae	6	0,0002	0,0006	0,0004
family	Comamonadaceae	7	0,0025	0,0009	0,0005
family	Comamonadaceae	8	0,0008	0,0004	0,0006
family	Comamonadaceae	9	0	0,0002	0,0005
family	Comamonadaceae	10	0,0004	0,0005	0,0008
family	Comamonadaceae	P	0,0034	0,0006	0,0005
family	Coriobacteriaceae	1	0,0015	0	0,0001
family	Coriobacteriaceae	2	0,001	0	0
family	Coriobacteriaceae	3	0,0003	0	0,0001
family	Coriobacteriaceae	4	0,0004	0,0001	0,0007

family	Coriobacteriaceae	5	0,001	0	0,0004
family	Coriobacteriaceae	6	0,0029	0	0
family	Coriobacteriaceae	7	0,0007	0	0
family	Coriobacteriaceae	8	0,0014	0	0,0001
family	Coriobacteriaceae	9	0,0008	0	0,0001
family	Coriobacteriaceae	10	0,0014	0	0,0001
family	Corynebacteriaceae	1	0,0087	0,0001	0
family	Corynebacteriaceae	2	0,0181	0	0
family	Corynebacteriaceae	3	0,2859	0,0006	0,0044
family	Corynebacteriaceae	4	0,1687	0,0028	0,0042
family	Corynebacteriaceae	5	0,0265	0,0004	0,0012
family	Corynebacteriaceae	6	0,008	0,0004	0,0003
family	Corynebacteriaceae	7	0,0331	0,0002	0,0001
family	Corynebacteriaceae	8	0,0136	0,0001	0,0002
family	Corynebacteriaceae	9	0,0251	0	0,0002
family	Corynebacteriaceae	10	0,0056	0,0001	0
family	Corynebacteriaceae	P	0,0016	0,0002	0,0001
family	Cytophagaceae	7	0	0	0,0001
family	Dermabacteraceae	7	0,0005	0	0
family	Enterobacteriaceae	1	0,0228	0,8429	0,8484
family	Enterobacteriaceae	2	0,0016	0,8561	0,8612
family	Enterobacteriaceae	3	0,0224	0,8646	0,8448
family	Enterobacteriaceae	4	0,0012	0,8221	0,5496
family	Enterobacteriaceae	5	0,0017	0,8802	0,7689
family	Enterobacteriaceae	6	0,0216	0,8967	0,8582
family	Enterobacteriaceae	7	0,0076	0,8727	0,8664
family	Enterobacteriaceae	8	0,0007	0,8941	0,8609
family	Enterobacteriaceae	9	0,0142	0,9044	0,8685
family	Enterobacteriaceae	10	0,0006	0,8925	0,839
family	Enterobacteriaceae	P	0,8461	0,8695	0,8827
family	Enterococcaceae	1	0	0,0055	0,0022
family	Enterococcaceae	2	0	0,0038	0,0019
family	Enterococcaceae	3	0	0,004	0,0021
family	Enterococcaceae	4	0,059	0,0023	0,0061
family	Enterococcaceae	5	0,0006	0,0022	0,003
family	Enterococcaceae	6	0	0,0018	0,0021
family	Enterococcaceae	7	0	0,0023	0,0031
family	Enterococcaceae	8	0	0,0034	0,0034
family	Enterococcaceae	9	0	0,0017	0,0022
family	Enterococcaceae	10	0,0025	0,0019	0,0011
family	Enterococcaceae	P	0,0096	0,0101	0,0108
family	Erysipelotrichaceae	1	0,0031	0	0
family	Erysipelotrichaceae	2	0	0	0,0001
family	Erysipelotrichaceae	8	0	0,0001	0
family	Fibrobacteraceae	1	0,0001	0	0
family	Flavobacteriaceae	1	0,0001	0,0001	0
family	Flavobacteriaceae	2	0,0048	0	0
family	Flavobacteriaceae	3	0	0,0001	0
family	Flavobacteriaceae	4	0,0005	0	0
family	Flavobacteriaceae	5	0,0001	0	0
family	Flavobacteriaceae	6	0,0006	0,0001	0
family	Flavobacteriaceae	7	0,0053	0,0001	0,0001
family	Flavobacteriaceae	8	0	0	0
family	Flavobacteriaceae	9	0,0025	0	0,0001
family	Flavobacteriaceae	10	0,0019	0	0

family	Fusobacteriaceae	3	0,0003	0,0001	0
family	Fusobacteriaceae	7	0,0025	0	0
family	Fusobacteriaceae	8	0,001	0	0
family	Fusobacteriaceae	10	0,0007	0	0
family	Hyphomicrobiaceae	1	0,0017	0	0
family	Hyphomicrobiaceae	3	0,0004	0	0
family	Hyphomicrobiaceae	6	0,001	0	0
family	Intrasporangiaceae	1	0,0001	0	0
family	Intrasporangiaceae	2	0,0019	0,0001	0
family	Intrasporangiaceae	3	0,0001	0	0
family	Intrasporangiaceae	7	0,0002	0	0
family	Intrasporangiaceae	9	0,0003	0	0
family	Lachnospiraceae	2	0,0006	0	0
family	Lachnospiraceae	3	0,0003	0	0
family	Lachnospiraceae	5	0,0001	0	0
family	Lachnospiraceae	6	0,0001	0	0
family	Lachnospiraceae	7	0,0021	0	0
family	Lachnospiraceae	9	0,0001	0	0
family	Lactobacillaceae	1	0,0022	0,0002	0
family	Lactobacillaceae	2	0,002	0,0001	0
family	Lactobacillaceae	3	0,0003	0	0
family	Lactobacillaceae	4	0,0009	0	0
family	Lactobacillaceae	5	0,0013	0,0002	0,0001
family	Lactobacillaceae	6	0,0086	0	0
family	Lactobacillaceae	7	0,0009	0	0
family	Lactobacillaceae	8	0,0078	0,0002	0
family	Lactobacillaceae	9	0,0038	0,0001	0
family	Lactobacillaceae	10	0,0032	0	0,0001
family	Leptotrichiaceae	5	0	0,0001	0
family	Leptotrichiaceae	7	0,0014	0	0
family	Leptotrichiaceae	10	0	0	0,0001
family	Leuconostocaceae	1	0,0023	0	0
family	Leuconostocaceae	3	0,001	0	0
family	Leuconostocaceae	6	0,002	0	0
family	Methanobacteriacea	4	0,0004	0	0
family	Methylobacteriaceae	2	0,0138	0	0
family	Methylobacteriaceae	4	0,0003	0	0
family	Methylobacteriaceae	6	0,0003	0	0
family	Methylobacteriaceae	7	0,0013	0	0
family	Methylobacteriaceae	9	0,0002	0	0
family	Methylobacteriaceae	10	0,0005	0	0
family	Microbacteriaceae	1	0,0009	0,0001	0,0001
family	Microbacteriaceae	2	0,0036	0,0002	0,0001
family	Microbacteriaceae	3	0,0001	0,0001	0
family	Microbacteriaceae	4	0	0	0,0001
family	Microbacteriaceae	5	0	0,0001	0
family	Microbacteriaceae	6	0,0001	0,0001	0,0001
family	Microbacteriaceae	7	0,0049	0	0,0001
family	Microbacteriaceae	8	0,0007	0,0001	0,0001
family	Microbacteriaceae	9	0,0001	0	0
family	Microbacteriaceae	10	0,0021	0	0
family	Microbacteriaceae	P	0,0001	0	0,0001
family	Micrococcaceae	1	0,0034	0,0008	0,0006
family	Micrococcaceae	2	0,0044	0,0006	0,0004
family	Micrococcaceae	3	0,0032	0,0007	0,0006

family	Micrococcaceae	4	0,0015	0,0006	0,0004
family	Micrococcaceae	5	0,0322	0,0008	0,0014
family	Micrococcaceae	6	0,0046	0,0006	0,0003
family	Micrococcaceae	7	0,0087	0,0006	0,0005
family	Micrococcaceae	8	0,0018	0,0003	0,0003
family	Micrococcaceae	9	0,0078	0,0003	0,0003
family	Micrococcaceae	10	0,0024	0,0005	0,0004
family	Micrococcaceae	P	0,0041	0,0004	0,0006
family	Mitochondria	4	0	0,0001	0
family	Mitochondria	6	0,0002	0	0
family	moorei	4	0,0002	0	0
family	moorei	10	0,0005	0	0
family	Moraxellaceae	1	0,0362	0,0077	0,0074
family	Moraxellaceae	2	0,1451	0,0061	0,0059
family	Moraxellaceae	3	0,0151	0,0084	0,0059
family	Moraxellaceae	4	0,027	0,0066	0,0042
family	Moraxellaceae	5	0,0047	0,0046	0,0044
family	Moraxellaceae	6	0,0617	0,0052	0,0049
family	Moraxellaceae	7	0,2679	0,0194	0,0065
family	Moraxellaceae	8	0,0674	0,0048	0,0071
family	Moraxellaceae	9	0,061	0,0047	0,0054
family	Moraxellaceae	10	0,0839	0,004	0,0037
family	Moraxellaceae	P	0,0072	0,0097	0,0059
family	Neisseriaceae	1	0,0005	0,0005	0,0001
family	Neisseriaceae	2	0,0036	0,0001	0,0001
family	Neisseriaceae	3	0,0001	0,0004	0,0001
family	Neisseriaceae	4	0,0001	0,0001	0,0018
family	Neisseriaceae	5	0	0,0002	0,0001
family	Neisseriaceae	6	0	0,0001	0,0001
family	Neisseriaceae	7	0,0039	0,0001	0,0001
family	Neisseriaceae	8	0,0003	0,0007	0,0015
family	Neisseriaceae	9	0	0	0,0001
family	Neisseriaceae	10	0,0013	0,0001	0,0001
family	Neisseriaceae	P	0,0007	0,0003	0,0001
family	Nevskiaceae	1	0	0,0001	0
family	Nevskiaceae	2	0,0004	0,0001	0,0001
family	Nevskiaceae	3	0,0003	0,0001	0,0001
family	Nevskiaceae	4	0,0001	0	0
family	Nevskiaceae	5	0,0003	0	0
family	Nevskiaceae	6	0,001	0	0,0001
family	Nevskiaceae	7	0,0017	0,0001	0,0001
family	Nevskiaceae	8	0,0004	0	0
family	Nevskiaceae	9	0	0	0
family	Nevskiaceae	10	0	0	0,0001
family	Nevskiaceae	P	0	0	0,0001
family	Nocardiaceae	3	0	0	0,0002
family	Nocardiaceae	5	0	0	0
family	Nocardiaceae	10	0	0	0,0001
family	Nocardiaceae	P	0,0001	0	0
family	Other	P	0,0001	0	0
family	Oxalobacteraceae	4	0,0003	0	0
family	Oxalobacteraceae	5	0,0001	0	0
family	Oxalobacteraceae	7	0	0	0,0001
family	Oxalobacteraceae	9	0,0007	0	0
family	Paenibacillaceae	1	0	0,0005	0,0002

family	Paenibacillaceae	2	0	0,0003	0,0002
family	Paenibacillaceae	3	0	0,0002	0,0001
family	Paenibacillaceae	4	0	0,0001	0,0002
family	Paenibacillaceae	5	0	0,0004	0,0002
family	Paenibacillaceae	6	0,0001	0,0001	0,0003
family	Paenibacillaceae	7	0	0,0001	0,0002
family	Paenibacillaceae	8	0	0,0001	0,0002
family	Paenibacillaceae	9	0	0,0003	0,0001
family	Paenibacillaceae	10	0,0001	0,0001	0,0002
family	Paenibacillaceae	P	0,0003	0,0003	0,0002
family	Pasteurellaceae	1	0,0041	0,0001	0,0002
family	Pasteurellaceae	2	0,0108	0,0002	0,0001
family	Pasteurellaceae	3	0,0014	0,0002	0,0002
family	Pasteurellaceae	4	0,0012	0	0,0002
family	Pasteurellaceae	5	0,0004	0,0004	0,0003
family	Pasteurellaceae	6	0,0048	0,0001	0,0003
family	Pasteurellaceae	7	0,0087	0,0001	0,0002
family	Pasteurellaceae	8	0,0033	0,0004	0,0012
family	Pasteurellaceae	9	0,0062	0,0001	0,0002
family	Pasteurellaceae	10	0,0156	0	0,0002
family	Pasteurellaceae	P	0,023	0,0002	0,0002
family	Peptostreptococcace	1	0,0006	0	0
family	Peptostreptococcace	2	0,0021	0	0
family	Peptostreptococcace	4	0,0012	0	0
family	Peptostreptococcace	7	0	0	0,0001
family	Peptostreptococcace	9	0,0027	0	0
family	Phyllobacteriaceae	4	0	0,0001	0
family	Phyllobacteriaceae	5	0,0004	0	0
family	Phyllobacteriaceae	6	0,0002	0,0001	0
family	Phyllobacteriaceae	7	0	0,0001	0
family	Phyllobacteriaceae	8	0	0	0,0001
family	Phyllobacteriaceae	9	0	0	0,0001
family	Phyllobacteriaceae	10	0	0	0,0001
family	Phyllobacteriaceae	P	0,0001	0,0001	0
family	Planococcaceae	1	0,0008	0	0
family	Planococcaceae	2	0,0027	0,0001	0
family	Planococcaceae	3	0,001	0	0
family	Planococcaceae	4	0,0007	0	0,0001
family	Planococcaceae	5	0,0001	0,0001	0,0001
family	Planococcaceae	6	0,0004	0,0001	0,0001
family	Planococcaceae	7	0,0003	0	0
family	Planococcaceae	8	0,0001	0	0
family	Planococcaceae	9	0,0001	0	0
family	Planococcaceae	10	0,0005	0	0
family	Planococcaceae	P	0	0,0001	0
family	Porphyromonadacea	1	0,0003	0	0
family	Porphyromonadacea	6	0	0	0,0001
family	Porphyromonadacea	8	0	0,0001	0
family	Porphyromonadacea	P	0,0002	0	0
family	Prevotellaceae	1	0	0	0,0004
family	Prevotellaceae	2	0,0026	0	0,0002
family	Prevotellaceae	3	0	0,0001	0,0001
family	Prevotellaceae	4	0,0002	0,0001	0,0001
family	Prevotellaceae	5	0,0011	0,0004	0,0001
family	Prevotellaceae	6	0	0,0001	0,0001

family	Prevotellaceae	7	0,0062	0	0,0001
family	Prevotellaceae	8	0,0003	0,0002	0,0002
family	Prevotellaceae	10	0	0,0002	0,0001
family	Prevotellaceae	P	0,0002	0,0001	0,0001
family	Propionibacteriaceae	1	0,0578	0,0002	0,0003
family	Propionibacteriaceae	2	0,0872	0,0004	0,0006
family	Propionibacteriaceae	3	0,03	0,0006	0,0002
family	Propionibacteriaceae	4	0,0229	0,0002	0,0002
family	Propionibacteriaceae	5	0,015	0,0008	0,0017
family	Propionibacteriaceae	6	0,1199	0,0033	0,0047
family	Propionibacteriaceae	7	0,1496	0,0017	0,0009
family	Propionibacteriaceae	8	0,0287	0,0011	0,002
family	Propionibacteriaceae	9	0,144	0,0012	0,0019
family	Propionibacteriaceae	10	0,064	0,0019	0,0008
family	Propionibacteriaceae	P	0,0101	0,0021	0,0005
family	Pseudoalteromonada	2	0,0002	0	0
family	Pseudoalteromonada	8	0,0002	0	0
family	Pseudomonadaceae	1	0,0135	0,1172	0,1023
family	Pseudomonadaceae	2	0,0523	0,1099	0,0874
family	Pseudomonadaceae	3	0,0076	0,1004	0,0748
family	Pseudomonadaceae	4	0,0066	0,0964	0,0636
family	Pseudomonadaceae	5	0,0007	0,0841	0,0679
family	Pseudomonadaceae	6	0,0401	0,077	0,0691
family	Pseudomonadaceae	7	0,0531	0,0841	0,1007
family	Pseudomonadaceae	8	0,0137	0,0755	0,0676
family	Pseudomonadaceae	9	0,0307	0,0779	0,0883
family	Pseudomonadaceae	10	0,0377	0,0679	0,0798
family	Pseudomonadaceae	P	0,0566	0,0841	0,0789
family	Pseudonocardiaceae	4	0,0002	0	0
family	Pseudonocardiaceae	10	0,0002	0	0
family	Rhizobiaceae	1	0	0,0006	0,0004
family	Rhizobiaceae	2	0,002	0,0004	0,0005
family	Rhizobiaceae	3	0,0025	0,0004	0,0005
family	Rhizobiaceae	4	0,0002	0,0005	0,0002
family	Rhizobiaceae	5	0,0005	0,0005	0,0002
family	Rhizobiaceae	6	0,003	0,0006	0,0004
family	Rhizobiaceae	7	0,0006	0,001	0,0004
family	Rhizobiaceae	8	0,0037	0,0004	0,0004
family	Rhizobiaceae	9	0,0024	0,0004	0,0005
family	Rhizobiaceae	10	0,0002	0,0002	0,0005
family	Rhizobiaceae	P	0,0007	0,0011	0,0005
family	Rhodobacteraceae	1	0,0014	0	0
family	Rhodobacteraceae	2	0,001	0	0
family	Rhodobacteraceae	3	0,001	0,0001	0
family	Rhodobacteraceae	4	0,0012	0	0
family	Rhodobacteraceae	5	0,0011	0	0
family	Rhodobacteraceae	6	0,0036	0	0,0001
family	Rhodobacteraceae	7	0,0067	0	0
family	Rhodobacteraceae	8	0,0127	0	0
family	Rhodobacteraceae	9	0,0024	0	0,0001
family	Rhodobacteraceae	10	0,0011	0	0
family	Rhodobacteraceae	P	0	0,0001	0
family	Rhodocyclaceae	4	0,0003	0	0
family	Rhodocyclaceae	6	0,0001	0	0,0001
family	Rikenellaceae	1	0,0002	0	0

family	Rikenellaceae	5	0,0003	0	0
family	Rikenellaceae	8	0,0003	0	0
family	Rikenellaceae	9	0	0	0
family	Rikenellaceae	P	0,0001	0	0
family	Ruminococcaceae	1	0,0009	0	0,0002
family	Ruminococcaceae	2	0,0012	0,0001	0
family	Ruminococcaceae	3	0,0003	0	0
family	Ruminococcaceae	4	0,0005	0,0002	0,0004
family	Ruminococcaceae	5	0,0015	0,0001	0,0003
family	Ruminococcaceae	6	0,0019	0,0001	0,0001
family	Ruminococcaceae	7	0,0085	0,0001	0,0001
family	Ruminococcaceae	8	0,0018	0,0001	0,0001
family	Ruminococcaceae	9	0,0038	0	0,0001
family	Ruminococcaceae	10	0,0018	0,0001	0,0001
family	Ruminococcaceae	P	0	0,0001	0,0001
family	Shewanellaceae	1	0	0,0006	0,0005
family	Shewanellaceae	2	0,0017	0,0008	0,0005
family	Shewanellaceae	3	0	0,0005	0,0002
family	Shewanellaceae	4	0	0,0007	0,0004
family	Shewanellaceae	5	0	0,0005	0,0001
family	Shewanellaceae	6	0	0,0005	0,0002
family	Shewanellaceae	7	0	0,0004	0,0004
family	Shewanellaceae	8	0,0016	0,0003	0,0003
family	Shewanellaceae	9	0	0,0002	0,0002
family	Shewanellaceae	10	0	0,0003	0,0004
family	Shewanellaceae	P	0,0002	0,0005	0,0004
family	Sphingomonadaceae	1	0,001	0,0001	0
family	Sphingomonadaceae	2	0,0014	0	0
family	Sphingomonadaceae	3	0,0002	0,0001	0,0001
family	Sphingomonadaceae	4	0,0008	0,0001	0
family	Sphingomonadaceae	6	0	0	0,0001
family	Sphingomonadaceae	7	0,0016	0,0002	0,0001
family	Sphingomonadaceae	8	0	0,0001	0,0001
family	Sphingomonadaceae	9	0,0001	0,0001	0,0001
family	Sphingomonadaceae	10	0,0013	0,0001	0
family	Sphingomonadaceae	P	0,0001	0,0002	0,0001
family	Staphylococcaceae	1	0,7773	0,0069	0,0234
family	Staphylococcaceae	2	0,5309	0,0104	0,032
family	Staphylococcaceae	3	0,2195	0,0069	0,0549
family	Staphylococcaceae	4	0,1941	0,0545	0,3537
family	Staphylococcaceae	5	0,4672	0,0096	0,1387
family	Staphylococcaceae	6	0,6577	0,0041	0,0511
family	Staphylococcaceae	7	0,318	0,0082	0,004
family	Staphylococcaceae	8	0,7499	0,0112	0,0431
family	Staphylococcaceae	9	0,6032	0,003	0,0241
family	Staphylococcaceae	10	0,7013	0,0228	0,0667
family	Staphylococcaceae	P	0,0068	0,0096	0,0083
family	Streptococcaceae	1	0,041	0,0112	0,0098
family	Streptococcaceae	2	0,0386	0,0072	0,0063
family	Streptococcaceae	3	0,0241	0,0064	0,006
family	Streptococcaceae	4	0,0156	0,0074	0,0086
family	Streptococcaceae	5	0,395	0,0106	0,0086
family	Streptococcaceae	6	0,0366	0,0063	0,005
family	Streptococcaceae	7	0,048	0,006	0,0112
family	Streptococcaceae	8	0,0363	0,0046	0,0081

family	Streptococcaceae	9	0,0398	0,004	0,005
family	Streptococcaceae	10	0,0416	0,0046	0,0042
family	Streptococcaceae	P	0,0141	0,0077	0,0069
family	Streptomycetaceae	1	0,0001	0	0
family	Streptomycetaceae	2	0,0001	0	0
family	Streptomycetaceae	3	0,0002	0	0
family	Streptomycetaceae	6	0,0004	0	0
family	Streptomycetaceae	7	0,0002	0	0
family	Streptomycetaceae	9	0,0003	0	0
family	Streptomycetaceae	10	0,0001	0	0
family	uncultured	2	0,0183	0	0
family	uncultured	3	0,0857	0,0002	0,0017
family	uncultured	4	0,035	0,0003	0,0004
family	uncultured	5	0,0052	0,0002	0,0001
family	uncultured	6	0,0011	0	0
family	uncultured	7	0,0021	0	0
family	uncultured	8	0,0342	0,0001	0,0001
family	uncultured	9	0,0094	0	0
family	uncultured	10	0,002	0	0
family	uncultured	P	0,0001	0	0
family	urartu	8	0,0004	0	0
family	Veillonellaceae	1	0,0016	0,0021	0,0013
family	Veillonellaceae	2	0,0051	0,0012	0,0008
family	Veillonellaceae	3	0,2656	0,0023	0,0014
family	Veillonellaceae	4	0,4504	0,003	0,0027
family	Veillonellaceae	5	0,0354	0,0015	0,0008
family	Veillonellaceae	6	0,0015	0,001	0,0004
family	Veillonellaceae	7	0,0181	0,0005	0,0027
family	Veillonellaceae	8	0	0,0004	0,0007
family	Veillonellaceae	9	0,009	0,0005	0,0006
family	Veillonellaceae	10	0,0034	0,0009	0,0005
family	Veillonellaceae	P	0,007	0,0011	0,0015
family	Verrucomicrobiaceae	4	0,0006	0	0
family	Vibrionaceae	1	0	0,0004	0,0002
family	Vibrionaceae	2	0	0,0003	0,0001
family	Vibrionaceae	3	0	0,0003	0,0002
family	Vibrionaceae	4	0	0,0002	0,0002
family	Vibrionaceae	5	0	0,0001	0
family	Vibrionaceae	6	0	0,0001	0,0002
family	Vibrionaceae	7	0	0,0001	0,0001
family	Vibrionaceae	8	0	0,0001	0,0001
family	Vibrionaceae	9	0	0	0,0002
family	Vibrionaceae	10	0	0,0001	0,0001
family	Vibrionaceae	P	0,0001	0,0002	0,0002
family	Xanthomonadaceae	1	0	0	0
family	Xanthomonadaceae	2	0,0034	0,0001	0
family	Xanthomonadaceae	4	0	0	0,0001
family	Xanthomonadaceae	5	0	0,0001	0
family	Xanthomonadaceae	6	0,0003	0,0001	0,0001
family	Xanthomonadaceae	7	0	0,0001	0
family	Xanthomonadaceae	8	0	0	0,0001
family	Xanthomonadaceae	9	0	0	0,0001
family	Xanthomonadaceae	P	0,0001	0,0001	0,0001
class	Actinobacteria	1	0,0715	0,0013	0,0011
class	Actinobacteria	2	0,134	0,0015	0,0013

class	Actinobacteria	3	0,4052	0,0022	0,0071
class	Actinobacteria	4	0,23	0,0041	0,0053
class	Actinobacteria	5	0,0797	0,0023	0,0044
class	Actinobacteria	6	0,1381	0,0044	0,0053
class	Actinobacteria	7	0,1994	0,0026	0,0018
class	Actinobacteria	8	0,0791	0,0018	0,0028
class	Actinobacteria	9	0,1892	0,0016	0,0025
class	Actinobacteria	10	0,0768	0,0026	0,0014
class	Actinobacteria	P	0,0195	0,0028	0,0013
class	Alphaproteobacteria	1	0,0046	0,0007	0,0006
class	Alphaproteobacteria	2	0,0216	0,0005	0,0005
class	Alphaproteobacteria	3	0,0051	0,0007	0,0006
class	Alphaproteobacteria	4	0,0033	0,0008	0,0003
class	Alphaproteobacteria	5	0,0022	0,0006	0,0003
class	Alphaproteobacteria	6	0,0082	0,0007	0,0007
class	Alphaproteobacteria	7	0,0117	0,0013	0,0006
class	Alphaproteobacteria	8	0,0182	0,0006	0,0007
class	Alphaproteobacteria	9	0,0069	0,0006	0,0009
class	Alphaproteobacteria	10	0,0049	0,0004	0,0005
class	Alphaproteobacteria	P	0,0011	0,0016	0,0007
class	Bacilli	1	0,8268	0,0256	0,0364
class	Bacilli	2	0,5797	0,0225	0,0409
class	Bacilli	3	0,2482	0,0185	0,0639
class	Bacilli	4	0,2709	0,0648	0,3698
class	Bacilli	5	0,8654	0,0242	0,1512
class	Bacilli	6	0,7086	0,013	0,0589
class	Bacilli	7	0,3779	0,017	0,0191
class	Bacilli	8	0,7977	0,02	0,0555
class	Bacilli	9	0,6499	0,0093	0,032
class	Bacilli	10	0,7558	0,03	0,0729
class	Bacilli	P	0,0337	0,0285	0,0268
class	Bacteroidia	1	0,0007	0,0001	0,0005
class	Bacteroidia	2	0,0031	0,0001	0,0002
class	Bacteroidia	3	0,0001	0,0001	0,0001
class	Bacteroidia	4	0,0002	0,0001	0,0001
class	Bacteroidia	5	0,0019	0,0004	0,0002
class	Bacteroidia	6	0,0003	0,0001	0,0002
class	Bacteroidia	7	0,0062	0,0001	0,0001
class	Bacteroidia	8	0,0008	0,0003	0,0002
class	Bacteroidia	9	0,0001	0,0001	0
class	Bacteroidia	10	0,0001	0,0003	0,0001
class	Bacteroidia	P	0,0007	0,0002	0,0001
class	Betaproteobacteria	1	0,0056	0,0008	0,0007
class	Betaproteobacteria	2	0,0187	0,0003	0,0007
class	Betaproteobacteria	3	0,0028	0,001	0,0003
class	Betaproteobacteria	4	0,0018	0,0004	0,002
class	Betaproteobacteria	5	0,0041	0,0004	0,0004
class	Betaproteobacteria	6	0,003	0,0007	0,0006
class	Betaproteobacteria	7	0,0162	0,001	0,0008
class	Betaproteobacteria	8	0,0027	0,0012	0,0022
class	Betaproteobacteria	9	0,0092	0,0003	0,0006
class	Betaproteobacteria	10	0,0097	0,0006	0,0009
class	Betaproteobacteria	P	0,0041	0,001	0,0006
class	Chloroplast	1	0,0029	0	0
class	Chloroplast	2	0,0037	0	0

class	Chloroplast	3	0,0014	0	0
class	Chloroplast	4	0,0014	0,0001	0
class	Chloroplast	5	0,0001	0,0001	0
class	Chloroplast	6	0,0019	0	0,0001
class	Chloroplast	7	0,0023	0,0001	0
class	Chloroplast	8	0,0007	0	0
class	Chloroplast	9	0,0039	0	0
class	Chloroplast	10	0,0018	0	0
class	Chloroplast	P	0,0001	0	0
class	Clostridia	1	0,0042	0,0001	0,0002
class	Clostridia	2	0,0126	0,0001	0
class	Clostridia	3	0,0241	0,0002	0
class	Clostridia	4	0,0039	0,0002	0,0005
class	Clostridia	5	0,002	0,0001	0,0003
class	Clostridia	6	0,0036	0,0001	0,0005
class	Clostridia	7	0,0136	0,0001	0,0002
class	Clostridia	8	0,0018	0,0001	0,0002
class	Clostridia	9	0,0129	0,0001	0,0001
class	Clostridia	10	0,0054	0,0001	0,0001
class	Clostridia	P	0,0001	0,0001	0,0002
class	Coriobacteriia	1	0,0015	0	0,0001
class	Coriobacteriia	2	0,001	0	0
class	Coriobacteriia	3	0,0003	0	0,0001
class	Coriobacteriia	4	0,0004	0,0001	0,0007
class	Coriobacteriia	5	0,001	0	0,0004
class	Coriobacteriia	6	0,0029	0	0
class	Coriobacteriia	7	0,0007	0	0
class	Coriobacteriia	8	0,0014	0	0,0001
class	Coriobacteriia	9	0,0008	0	0,0001
class	Coriobacteriia	10	0,0014	0	0,0001
class	Cytophagia	7	0	0	0,0001
class	Epsilonproteobacteri	7	0,0033	0	0
class	Erysipelotrichia	1	0,0031	0	0
class	Erysipelotrichia	2	0	0	0,0001
class	Erysipelotrichia	8	0	0,0001	0
class	Fibrobacteria	1	0,0001	0	0
class	Flavobacteriia	1	0,0001	0,0001	0
class	Flavobacteriia	2	0,0048	0	0
class	Flavobacteriia	3	0	0,0001	0
class	Flavobacteriia	4	0,0005	0	0
class	Flavobacteriia	5	0,0001	0	0
class	Flavobacteriia	6	0,0006	0,0001	0
class	Flavobacteriia	7	0,0053	0,0001	0,0001
class	Flavobacteriia	8	0	0	0
class	Flavobacteriia	9	0,0025	0	0,0001
class	Flavobacteriia	10	0,0019	0	0
class	Fusobacteriia	3	0,0003	0,0001	0
class	Fusobacteriia	5	0	0,0001	0
class	Fusobacteriia	7	0,0039	0	0
class	Fusobacteriia	8	0,001	0	0
class	Fusobacteriia	10	0,0007	0	0,0001
class	Gammaproteobacter	1	0,0775	0,9692	0,9592
class	Gammaproteobacter	2	0,2155	0,9736	0,9554
class	Gammaproteobacter	3	0,0468	0,9748	0,9264
class	Gammaproteobacter	4	0,0361	0,9263	0,6185

class	Gammaproteobacter	5	0,0078	0,9701	0,8419
class	Gammaproteobacter	6	0,131	0,9799	0,9333
class	Gammaproteobacter	7	0,3412	0,9772	0,9744
class	Gammaproteobacter	8	0,0964	0,9754	0,9376
class	Gammaproteobacter	9	0,1123	0,9876	0,9631
class	Gammaproteobacter	10	0,1381	0,9651	0,9233
class	Gammaproteobacter	P	0,9335	0,9646	0,9686
class	Methanobacteria	4	0,0004	0	0
class	Negativicutes	1	0,0016	0,0021	0,0013
class	Negativicutes	2	0,0051	0,0012	0,0008
class	Negativicutes	3	0,2656	0,0023	0,0014
class	Negativicutes	4	0,4504	0,003	0,0027
class	Negativicutes	5	0,0356	0,0016	0,0008
class	Negativicutes	6	0,0015	0,001	0,0004
class	Negativicutes	7	0,0181	0,0005	0,0027
class	Negativicutes	8	0	0,0004	0,0007
class	Negativicutes	9	0,009	0,0005	0,0006
class	Negativicutes	10	0,0034	0,0009	0,0006
class	Negativicutes	P	0,007	0,0011	0,0015
class	Sphingobacteriia	9	0,0032	0	0
class	Thermoleophilia	3	0,0001	0	0
class	Thermoleophilia	6	0,0002	0	0
class	Thermomicrobia	2	0	0	0,0001
class	Verrucomicrobiae	4	0,0006	0	0
order	90	6	0,0001	0	0
order	Actinomycetales	1	0,0006	0,0001	0
order	Actinomycetales	2	0,0004	0,0001	0,0001
order	Actinomycetales	4	0,0015	0,0001	0
order	Actinomycetales	5	0,0003	0,0001	0
order	Actinomycetales	6	0,0006	0	0
order	Actinomycetales	7	0	0	0,0001
order	Actinomycetales	8	0	0	0,0001
order	Actinomycetales	9	0	0	0,0001
order	Actinomycetales	10	0,0003	0	0
order	Actinomycetales	P	0,0033	0	0,0001
order	Aeromonadales	6	0,0001	0	0
order	Aeromonadales	8	0,0052	0	0
order	Aeromonadales	10	0,0002	0	0
order	Aeromonadales	P	0	0,0001	0
order	Alteromonadales	1	0	0,0006	0,0005
order	Alteromonadales	2	0,0019	0,0008	0,0005
order	Alteromonadales	3	0	0,0005	0,0002
order	Alteromonadales	4	0	0,0007	0,0004
order	Alteromonadales	5	0	0,0005	0,0001
order	Alteromonadales	6	0	0,0005	0,0002
order	Alteromonadales	7	0	0,0004	0,0004
order	Alteromonadales	8	0,0018	0,0003	0,0003
order	Alteromonadales	9	0	0,0002	0,0002
order	Alteromonadales	10	0	0,0003	0,0004
order	Alteromonadales	P	0,0002	0,0005	0,0004
order	B38	1	0	0,0002	0,0001
order	B38	2	0	0,0001	0,0001
order	B38	3	0	0,0002	0,0002
order	B38	4	0	0,0001	0,0001
order	B38	5	0	0,0001	0,0002

order	B38	6	0,0013	0,0001	0,0002
order	B38	7	0,0001	0,0001	0,0002
order	B38	8	0,0039	0,0001	0,0002
order	B38	9	0,0002	0,0002	0,0001
order	B38	10	0,0001	0,0002	0,0001
order	B38	P	0,0001	0,0002	0,0001
order	Bacillales	1	0,7807	0,0081	0,0239
order	Bacillales	2	0,5373	0,0112	0,0325
order	Bacillales	3	0,2217	0,0075	0,0553
order	Bacillales	4	0,1954	0,055	0,3549
order	Bacillales	5	0,4681	0,0108	0,1393
order	Bacillales	6	0,6608	0,0046	0,0517
order	Bacillales	7	0,329	0,0084	0,0044
order	Bacillales	8	0,7522	0,0116	0,0438
order	Bacillales	9	0,6042	0,0034	0,0246
order	Bacillales	10	0,7052	0,0232	0,0672
order	Bacillales	P	0,009	0,0103	0,0089
order	bacterium	P	0,0001	0	0
order	Bacteroidales	1	0,0007	0,0001	0,0005
order	Bacteroidales	2	0,0031	0,0001	0,0002
order	Bacteroidales	3	0,0001	0,0001	0,0001
order	Bacteroidales	4	0,0002	0,0001	0,0001
order	Bacteroidales	5	0,0019	0,0004	0,0002
order	Bacteroidales	6	0,0003	0,0001	0,0002
order	Bacteroidales	7	0,0062	0,0001	0,0001
order	Bacteroidales	8	0,0008	0,0003	0,0002
order	Bacteroidales	9	0,0001	0,0001	0
order	Bacteroidales	10	0,0001	0,0003	0,0001
order	Bacteroidales	P	0,0007	0,0002	0,0001
order	Bifidobacteriales	5	0,0001	0	0
order	Bifidobacteriales	6	0,0018	0	0
order	Bifidobacteriales	9	0,0005	0	0
order	Burkholderiales	1	0,0051	0,0003	0,0006
order	Burkholderiales	2	0,015	0,0002	0,0006
order	Burkholderiales	3	0,0026	0,0007	0,0002
order	Burkholderiales	4	0,0015	0,0003	0,0002
order	Burkholderiales	5	0,0041	0,0002	0,0003
order	Burkholderiales	6	0,0029	0,0006	0,0004
order	Burkholderiales	7	0,0123	0,0009	0,0007
order	Burkholderiales	8	0,0024	0,0005	0,0007
order	Burkholderiales	9	0,0092	0,0003	0,0005
order	Burkholderiales	10	0,0084	0,0005	0,0008
order	Burkholderiales	P	0,0034	0,0007	0,0005
order	Campylobacterales	7	0,0033	0	0
order	Cardiobacteriales	7	0,0019	0	0
order	Cardiobacteriales	P	0,0001	0	0
order	Caulobacterales	2	0,0029	0	0
order	Caulobacterales	5	0	0,0001	0
order	Caulobacterales	7	0	0	0,0001
order	Caulobacterales	8	0,0019	0	0
order	Caulobacterales	9	0,0015	0	0
order	Caulobacterales	10	0,0018	0	0
order	Caulobacterales	P	0	0,0001	0
order	Clostridiales	1	0,0042	0,0001	0,0002
order	Clostridiales	2	0,0126	0,0001	0

order	Clostridiales	3	0,0241	0,0002	0
order	Clostridiales	4	0,0039	0,0002	0,0005
order	Clostridiales	5	0,002	0,0001	0,0003
order	Clostridiales	6	0,0036	0,0001	0,0005
order	Clostridiales	7	0,0136	0,0001	0,0002
order	Clostridiales	8	0,0018	0,0001	0,0002
order	Clostridiales	9	0,0129	0,0001	0,0001
order	Clostridiales	10	0,0054	0,0001	0,0001
order	Clostridiales	P	0,0001	0,0001	0,0002
order	Coriobacteriales	1	0,0015	0	0,0001
order	Coriobacteriales	2	0,001	0	0
order	Coriobacteriales	3	0,0003	0	0,0001
order	Coriobacteriales	4	0,0004	0,0001	0,0007
order	Coriobacteriales	5	0,001	0	0,0004
order	Coriobacteriales	6	0,0029	0	0
order	Coriobacteriales	7	0,0007	0	0
order	Coriobacteriales	8	0,0014	0	0,0001
order	Coriobacteriales	9	0,0008	0	0,0001
order	Coriobacteriales	10	0,0014	0	0,0001
order	Corynebacteriales	1	0,0087	0,0001	0
order	Corynebacteriales	2	0,0364	0,0001	0
order	Corynebacteriales	3	0,3716	0,0007	0,0063
order	Corynebacteriales	4	0,2038	0,0032	0,0046
order	Corynebacteriales	5	0,0318	0,0006	0,0013
order	Corynebacteriales	6	0,0092	0,0004	0,0003
order	Corynebacteriales	7	0,0352	0,0002	0,0002
order	Corynebacteriales	8	0,0478	0,0002	0,0004
order	Corynebacteriales	9	0,0346	0,0001	0,0002
order	Corynebacteriales	10	0,0076	0,0001	0,0001
order	Corynebacteriales	P	0,0018	0,0002	0,0001
order	Cytophagales	7	0	0	0,0001
order	Enterobacteriales	1	0,0228	0,8429	0,8484
order	Enterobacteriales	2	0,0016	0,8561	0,8612
order	Enterobacteriales	3	0,0224	0,8646	0,8448
order	Enterobacteriales	4	0,0012	0,8221	0,5496
order	Enterobacteriales	5	0,0017	0,8802	0,7689
order	Enterobacteriales	6	0,0216	0,8967	0,8582
order	Enterobacteriales	7	0,0076	0,8727	0,8664
order	Enterobacteriales	8	0,0007	0,8941	0,8609
order	Enterobacteriales	9	0,0142	0,9044	0,8685
order	Enterobacteriales	10	0,0006	0,8925	0,839
order	Enterobacteriales	P	0,8461	0,8695	0,8827
order	Erysipelotrichales	1	0,0031	0	0
order	Erysipelotrichales	2	0	0	0,0001
order	Erysipelotrichales	8	0	0,0001	0
order	Fibrobacterales	1	0,0001	0	0
order	Flavobacteriales	1	0,0001	0,0001	0
order	Flavobacteriales	2	0,0048	0	0
order	Flavobacteriales	3	0	0,0001	0
order	Flavobacteriales	4	0,0005	0	0
order	Flavobacteriales	5	0,0001	0	0
order	Flavobacteriales	6	0,0006	0,0001	0
order	Flavobacteriales	7	0,0053	0,0001	0,0001
order	Flavobacteriales	8	0	0	0
order	Flavobacteriales	9	0,0025	0	0,0001

order	Flavobacteriales	10	0,0019	0	0
order	Fusobacteriales	3	0,0003	0,0001	0
order	Fusobacteriales	5	0	0,0001	0
order	Fusobacteriales	7	0,0039	0	0
order	Fusobacteriales	8	0,001	0	0
order	Fusobacteriales	10	0,0007	0	0,0001
order	Lactobacillales	1	0,0461	0,0175	0,0125
order	Lactobacillales	2	0,0424	0,0113	0,0083
order	Lactobacillales	3	0,0265	0,011	0,0086
order	Lactobacillales	4	0,0755	0,0098	0,0149
order	Lactobacillales	5	0,3973	0,0133	0,0119
order	Lactobacillales	6	0,0478	0,0084	0,0072
order	Lactobacillales	7	0,0489	0,0086	0,0146
order	Lactobacillales	8	0,0455	0,0084	0,0117
order	Lactobacillales	9	0,0458	0,0059	0,0073
order	Lactobacillales	10	0,0506	0,0068	0,0056
order	Lactobacillales	P	0,0246	0,0181	0,0179
order	Methanobacteriales	4	0,0004	0	0
order	Micrococcales	1	0,0044	0,0009	0,0007
order	Micrococcales	2	0,0099	0,0009	0,0005
order	Micrococcales	3	0,0034	0,0008	0,0006
order	Micrococcales	4	0,0016	0,0007	0,0005
order	Micrococcales	5	0,0325	0,0009	0,0014
order	Micrococcales	6	0,0062	0,0007	0,0004
order	Micrococcales	7	0,0143	0,0007	0,0007
order	Micrococcales	8	0,0025	0,0005	0,0004
order	Micrococcales	9	0,0099	0,0003	0,0003
order	Micrococcales	10	0,0046	0,0005	0,0004
order	Micrococcales	P	0,0042	0,0004	0,0006
order	Neisseriales	1	0,0005	0,0005	0,0001
order	Neisseriales	2	0,0036	0,0001	0,0001
order	Neisseriales	3	0,0001	0,0004	0,0001
order	Neisseriales	4	0,0001	0,0001	0,0018
order	Neisseriales	5	0	0,0002	0,0001
order	Neisseriales	6	0	0,0001	0,0001
order	Neisseriales	7	0,0039	0,0001	0,0001
order	Neisseriales	8	0,0003	0,0007	0,0015
order	Neisseriales	9	0	0	0,0001
order	Neisseriales	10	0,0013	0,0001	0,0001
order	Neisseriales	P	0,0007	0,0003	0,0001
order	Pasteurellales	1	0,0041	0,0001	0,0002
order	Pasteurellales	2	0,0108	0,0002	0,0001
order	Pasteurellales	3	0,0014	0,0002	0,0002
order	Pasteurellales	4	0,0012	0	0,0002
order	Pasteurellales	5	0,0004	0,0004	0,0003
order	Pasteurellales	6	0,0048	0,0001	0,0003
order	Pasteurellales	7	0,0087	0,0001	0,0002
order	Pasteurellales	8	0,0033	0,0004	0,0012
order	Pasteurellales	9	0,0062	0,0001	0,0002
order	Pasteurellales	10	0,0156	0	0,0002
order	Pasteurellales	P	0,023	0,0002	0,0002
order	Propionibacteriales	1	0,0578	0,0002	0,0003
order	Propionibacteriales	2	0,0872	0,0004	0,0006
order	Propionibacteriales	3	0,03	0,0006	0,0002
order	Propionibacteriales	4	0,0229	0,0002	0,0002

order	Propionibacteriales	5	0,015	0,0008	0,0017
order	Propionibacteriales	6	0,1199	0,0033	0,0047
order	Propionibacteriales	7	0,1496	0,0017	0,0009
order	Propionibacteriales	8	0,0287	0,0011	0,002
order	Propionibacteriales	9	0,144	0,0012	0,0019
order	Propionibacteriales	10	0,064	0,0019	0,0008
order	Propionibacteriales	P	0,0101	0,0021	0,0005
order	Pseudomonadales	1	0,0497	0,1249	0,1097
order	Pseudomonadales	2	0,1974	0,116	0,0933
order	Pseudomonadales	3	0,0227	0,1088	0,0807
order	Pseudomonadales	4	0,0336	0,103	0,0678
order	Pseudomonadales	5	0,0054	0,0887	0,0723
order	Pseudomonadales	6	0,1018	0,0822	0,074
order	Pseudomonadales	7	0,3211	0,1035	0,1071
order	Pseudomonadales	8	0,0811	0,0803	0,0747
order	Pseudomonadales	9	0,0917	0,0826	0,0938
order	Pseudomonadales	10	0,1216	0,0719	0,0835
order	Pseudomonadales	P	0,0638	0,0939	0,0849
order	Pseudonocardiales	4	0,0002	0	0
order	Pseudonocardiales	10	0,0002	0	0
order	Rhizobiales	1	0,0021	0,0006	0,0005
order	Rhizobiales	2	0,0163	0,0005	0,0005
order	Rhizobiales	3	0,004	0,0005	0,0005
order	Rhizobiales	4	0,0008	0,0006	0,0002
order	Rhizobiales	5	0,001	0,0005	0,0002
order	Rhizobiales	6	0,0044	0,0007	0,0004
order	Rhizobiales	7	0,0034	0,0011	0,0004
order	Rhizobiales	8	0,0037	0,0004	0,0005
order	Rhizobiales	9	0,0029	0,0005	0,0007
order	Rhizobiales	10	0,0007	0,0003	0,0005
order	Rhizobiales	P	0,0009	0,0012	0,0005
order	Rhodobacterales	1	0,0014	0	0
order	Rhodobacterales	2	0,001	0	0
order	Rhodobacterales	3	0,001	0,0001	0
order	Rhodobacterales	4	0,0012	0	0
order	Rhodobacterales	5	0,0011	0	0
order	Rhodobacterales	6	0,0036	0	0,0001
order	Rhodobacterales	7	0,0067	0	0
order	Rhodobacterales	8	0,0127	0	0
order	Rhodobacterales	9	0,0024	0	0,0001
order	Rhodobacterales	10	0,0011	0	0
order	Rhodobacterales	P	0	0,0001	0
order	Rhodocyclales	4	0,0003	0	0
order	Rhodocyclales	6	0,0001	0	0,0001
order	Rhodospirillales	4	0,0005	0	0
order	Rickettsiales	3	0	0,0001	0
order	Rickettsiales	4	0	0,0001	0
order	Rickettsiales	6	0,0002	0	0
order	Selenomonadales	1	0,0016	0,0021	0,0013
order	Selenomonadales	2	0,0051	0,0012	0,0008
order	Selenomonadales	3	0,2656	0,0023	0,0014
order	Selenomonadales	4	0,4504	0,003	0,0027
order	Selenomonadales	5	0,0356	0,0016	0,0008
order	Selenomonadales	6	0,0015	0,001	0,0004
order	Selenomonadales	7	0,0181	0,0005	0,0027

order	Selenomonadales	8	0	0,0004	0,0007
order	Selenomonadales	9	0,009	0,0005	0,0006
order	Selenomonadales	10	0,0034	0,0009	0,0006
order	Selenomonadales	P	0,007	0,0011	0,0015
order	Solirubrobacterales	3	0,0001	0	0
order	Solirubrobacterales	6	0,0002	0	0
order	Sphingobacteriales	9	0,0032	0	0
order	Sphingomonadales	1	0,001	0,0001	0
order	Sphingomonadales	2	0,0014	0	0
order	Sphingomonadales	3	0,0002	0,0001	0,0001
order	Sphingomonadales	4	0,0008	0,0001	0
order	Sphingomonadales	6	0	0	0,0001
order	Sphingomonadales	7	0,0016	0,0002	0,0001
order	Sphingomonadales	8	0	0,0001	0,0001
order	Sphingomonadales	9	0,0001	0,0001	0,0001
order	Sphingomonadales	10	0,0013	0,0001	0
order	Sphingomonadales	P	0,0001	0,0002	0,0001
order	Streptomycetales	1	0,0001	0	0
order	Streptomycetales	2	0,0001	0	0
order	Streptomycetales	3	0,0002	0	0
order	Streptomycetales	6	0,0004	0	0
order	Streptomycetales	7	0,0002	0	0
order	Streptomycetales	9	0,0003	0	0
order	Streptomycetales	10	0,0001	0	0
order	Verrucomicrobiales	4	0,0006	0	0
order	Vibrionales	1	0	0,0004	0,0002
order	Vibrionales	2	0	0,0003	0,0001
order	Vibrionales	3	0	0,0003	0,0002
order	Vibrionales	4	0	0,0002	0,0002
order	Vibrionales	5	0	0,0001	0
order	Vibrionales	6	0	0,0001	0,0002
order	Vibrionales	7	0	0,0001	0,0001
order	Vibrionales	8	0	0,0001	0,0001
order	Vibrionales	9	0	0	0,0002
order	Vibrionales	10	0	0,0001	0,0001
order	Vibrionales	P	0,0001	0,0002	0,0002
order	Xanthomonadales	1	0,0009	0,0001	0,0001
order	Xanthomonadales	2	0,0038	0,0001	0,0001
order	Xanthomonadales	3	0,0003	0,0001	0,0001
order	Xanthomonadales	4	0,0001	0,0001	0,0001
order	Xanthomonadales	5	0,0003	0,0002	0,0001
order	Xanthomonadales	6	0,0013	0,0001	0,0002
order	Xanthomonadales	7	0,0017	0,0002	0,0001
order	Xanthomonadales	8	0,0004	0,0001	0,0001
order	Xanthomonadales	9	0	0,0001	0,0001
order	Xanthomonadales	10	0	0,0001	0,0001
order	Xanthomonadales	P	0,0001	0,0001	0,0002
phylum	Actinobacteria	1	0,073	0,0013	0,0011
phylum	Actinobacteria	2	0,1351	0,0015	0,0013
phylum	Actinobacteria	3	0,4055	0,0022	0,0072
phylum	Actinobacteria	4	0,2304	0,0042	0,006
phylum	Actinobacteria	5	0,0807	0,0024	0,0048
phylum	Actinobacteria	6	0,1412	0,0044	0,0054
phylum	Actinobacteria	7	0,2001	0,0026	0,0018
phylum	Actinobacteria	8	0,0805	0,0019	0,0029

phylum Actinobacteria	9	0,19	0,0016	0,0025
phylum Actinobacteria	10	0,0782	0,0026	0,0015
phylum Actinobacteria	P	0,0195	0,0028	0,0013
phylum Bacteroidetes	1	0,0007	0,0001	0,0005
phylum Bacteroidetes	2	0,0079	0,0001	0,0002
phylum Bacteroidetes	3	0,0001	0,0001	0,0001
phylum Bacteroidetes	4	0,0007	0,0002	0,0001
phylum Bacteroidetes	5	0,002	0,0004	0,0002
phylum Bacteroidetes	6	0,001	0,0002	0,0002
phylum Bacteroidetes	7	0,0115	0,0002	0,0003
phylum Bacteroidetes	8	0,0008	0,0003	0,0002
phylum Bacteroidetes	9	0,0058	0,0001	0,0001
phylum Bacteroidetes	10	0,002	0,0003	0,0001
phylum Bacteroidetes	P	0,0007	0,0002	0,0002
phylum Chloroflexi	2	0	0	0,0001
phylum Cyanobacteria	1	0,0029	0	0
phylum Cyanobacteria	2	0,0037	0	0
phylum Cyanobacteria	3	0,0014	0	0
phylum Cyanobacteria	4	0,0014	0,0001	0
phylum Cyanobacteria	5	0,0001	0,0001	0
phylum Cyanobacteria	6	0,0019	0	0,0001
phylum Cyanobacteria	7	0,0023	0,0001	0
phylum Cyanobacteria	8	0,0007	0	0
phylum Cyanobacteria	9	0,0039	0	0
phylum Cyanobacteria	10	0,0018	0	0
phylum Cyanobacteria	P	0,0001	0	0
phylum Euryarchaeota	4	0,0004	0	0
phylum Fibrobacteres	1	0,0001	0	0
phylum Firmicutes	1	0,8357	0,0278	0,0379
phylum Firmicutes	2	0,5974	0,0238	0,0417
phylum Firmicutes	3	0,5379	0,021	0,0654
phylum Firmicutes	4	0,7252	0,068	0,373
phylum Firmicutes	5	0,9031	0,0258	0,1524
phylum Firmicutes	6	0,7137	0,0141	0,0598
phylum Firmicutes	7	0,4097	0,0176	0,022
phylum Firmicutes	8	0,7996	0,0207	0,0563
phylum Firmicutes	9	0,6718	0,0099	0,0327
phylum Firmicutes	10	0,7646	0,0309	0,0736
phylum Firmicutes	P	0,0408	0,0297	0,0285
phylum Fusobacteria	3	0,0003	0,0001	0
phylum Fusobacteria	5	0	0,0001	0
phylum Fusobacteria	7	0,0039	0	0
phylum Fusobacteria	8	0,001	0	0
phylum Fusobacteria	10	0,0007	0	0,0001
phylum Proteobacteria	1	0,0876	0,9707	0,9604
phylum Proteobacteria	2	0,2558	0,9745	0,9566
phylum Proteobacteria	3	0,0547	0,9766	0,9273
phylum Proteobacteria	4	0,0413	0,9275	0,6208
phylum Proteobacteria	5	0,0141	0,9712	0,8426
phylum Proteobacteria	6	0,1422	0,9813	0,9345
phylum Proteobacteria	7	0,3725	0,9795	0,9759
phylum Proteobacteria	8	0,1174	0,9771	0,9405
phylum Proteobacteria	9	0,1284	0,9884	0,9646
phylum Proteobacteria	10	0,1527	0,9661	0,9248
phylum Proteobacteria	P	0,9386	0,9671	0,9699

phylum	Saccharibacteria	P	0,0001	0	0
phylum	Verrucomicrobia	4	0,0006	0	0
